# TEAD transcription factors are required for normal primary myoblast differentiation *in vitro* and muscle regeneration *in vivo*

**DOI:** 10.1371/journal.pgen.1006600

**Published:** 2017-02-08

**Authors:** Shilpy Joshi, Guillaume Davidson, Stéphanie Le Gras, Shuichi Watanabe, Thomas Braun, Gabrielle Mengus, Irwin Davidson

**Affiliations:** 1 Department of Functional Genomics and Cancer, Institut de Génétique et de Biologie Moléculaire et Cellulaire, CNRS/INSERM/UNISTRA, Illkirch, France; 2 Department of Cardiac Development and Remodeling, Max-Planck-Institute for Heart and Lung Research, Ludwigstrasse, Bad Nauheim, Germany; Stanford University School of Medicine, UNITED STATES

## Abstract

The TEAD family of transcription factors (TEAD1-4) bind the MCAT element in the regulatory elements of both growth promoting and myogenic differentiation genes. Defining TEAD transcription factor function in myogenesis has proved elusive due to overlapping expression of family members and their functional redundancy. We show that silencing of either *Tead1*, *Tead2* or *Tead4* did not effect primary myoblast (PM) differentiation, but that their simultaneous knockdown strongly impaired differentiation. In contrast, *Tead1* or *Tead4* silencing impaired C2C12 differentiation showing their different contributions in PMs and C2C12 cells. Chromatin immunoprecipitation identified enhancers associated with myogenic genes bound by combinations of Tead4, Myod1 or Myog. Tead4 regulated distinct gene sets in C2C12 cells and PMs involving both activation of the myogenic program and repression of growth and signaling pathways. ChIP-seq from mature mouse muscle fibres *in vivo* identified a set of highly transcribed muscle cell-identity genes and sites bound by Tead1 and Tead4. Although inactivation of Tead4 in mature muscle fibres caused no obvious phenotype under normal conditions, notexin-induced muscle regeneration was delayed in Tead4 mutants suggesting an important role in myogenic differentiation *in vivo*. By combining knockdown in cell models *in vitro* with Tead4 inactivation in muscle *in vivo*, we provide the first comprehensive description of the specific and redundant roles of Tead factors in myogenic differentiation.

## Author summary

Aspects of muscle differentiation can be reproduced using the C2C12 cell line or primary myoblasts both of which can be differentiated to form myotubes *in vitro*. While the functions of recognised myogenic proteins such as Myogenin, Myod1 and MEF-family transcription factors in this process have been extensively studied, the role of the Tead factors has received only limited attention. Tead factors have well defined roles as mediators of Hippo signalling in promoting cell growth and oncogenic transformation, but are also involved in myogenic differentiation involving cell cycle arrest and activation of the myogenic gene expression program. Using integrative genomics and knockdowns in cell based models, we show that Tead factors are essential for differentiation of C2C12 cells and primary myoblasts, but make different contributions activating a distinct set of myogenesis genes in each cell type. We also developped effective chromatin immunoprecipitation from mature mouse muscle fibres allowing identification of highly transcribed muscle identify genes and identification of Tead1 and Tead4 occupied sites. Somatic inactivation *in vivo* revealed an important role for Tead4 in muscle fibre regeneration. The integration of genomics and loss of function in cell models *in vitro* and muscle *in vivo* provide the first comprehensive description Tead factors in myogenic differentiation.

## Introduction

The TEAD(1–4) transcription factors [1, 2] bind to a consensus MCAT (5’-CATTCCA/T-3’) element, originally identified as the SV40 enhancer GT-II motif [3] [4–6], through the evolutionarily conserved TEA/ATTS DNA binding domain [7, 8]. Mammalian TEADs are widely expressed with prominent Tead1 and Tead4 expression in skeletal muscle, lung, heart and nervous system. Tead factors act as mediators of the Hippo signalling pathway interacting with the Yap and Wwtr1 (Taz) transcriptional co-activators to regulate proliferation, oncogenesis, stem cell maintenance and differentiation and control of organ size [9–14].

Teads also play an important role in skeletal, cardiac, and smooth muscle differentiation and physiology [15–18]. Tead4 is expressed in developing skeletal muscle in mouse embryos [2] and at later stages both Tead1 and Tead4 are co-expressed and co-localise to somites [19]. Blais et al. showed that Myod1 and Myog directly bind the *Tead4* promoter and activate its expression during C2C12 cell differentiation *in vitro* [20]. Subsequently, we showed that stable shRNA-mediated Tead4 knockdown led to formation of shortened C2C12 myotubes [21]. ChIP-chip experiments in C2C12 cells overexpressing Flag-HA-Tead4 revealed that Tead4 occupied 867 promoters including *Myog*, and *Cav3*. RNA-seq identified a set of genes down-regulated upon Tead4 knockdown amongst which are muscle structural and regulatory proteins. While our data described a role for Tead4 in activating muscle genes during differentiation, we also suggested that Tead4 may repress *Ctgf* and *Ccnd1* expression contributing to cell cycle exit. However, we did not specifically address the role of other Teads in these cells.

Here, we show by siRNA silencing that Tead factors are essential for primary myoblast (PM) differentiation, but that Tead1, Tead2 or Tead4 play partially redundant roles. In contrast to C2C12 cells, silencing of Tead1 or Tead4 both impaired differentiation indicating a differential requirement for these factors in PMs and C2C12 cells. ChIP-seq of Tead1 and Tead4 identified their binding sites C2C12 cells and in mature muscle fibres. RNA-seq identified distinct but overlapping sets of genes deregulated by Tead silencing in C2C12 cells and PMs. Furthermore, somatic inactivation in muscle *in vivo* revealed an important role for Tead4 in muscle regneration. We therefore provide the first comprehensive study of the specific and redundant regulatory roles of Tead factors in myogenic differentiation.

## Results

### Tead factors are essential for primary myoblast differentiation

**Fig 1:**
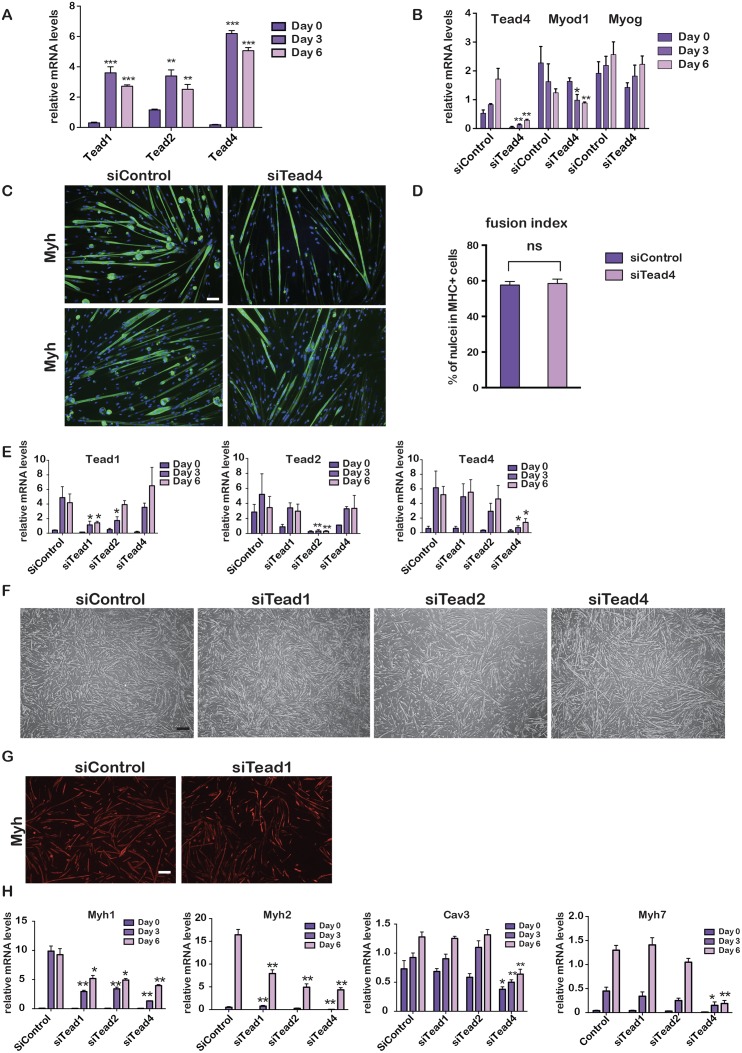
Tead factors in PMs. **A-B**. Gene expression was quantified by RT-qPCR in differentiating PMs after transfection with the indicated siRNAs. **C.** Fluorescence microscopy images after 6 days of differentiation of PMs transfected with the indicated siRNAs. Green channel shows staining with Myh antibody, and blue Dapi-stained nuclei. 10X magnification. Scale bar 100 μm. **D.** Fusion index of siControl and *siTead4* cells. **E.** Quantification of gene expression during PM differentiation after transfection with the indicated siRNAs. **F.** Bright field microscopy images after 6 days of differentiation of cells transfected with the indicated siRNAs. Scale bar 200 μm **G**. Fluorescence microscopy images of Myh-staining after 6 days of differentiation following transfection of the indicated siRNAs. Scale bar 200 μm. **H** Quantification of gene expression after transfection with the indicated siRNAs. In panels A, B, E and H data was analysed by multiple t-tests. p-value *** <0,0001 p-value ** <0,001, p-value * <0,01. In panel A, p-value is with respect to day 0 for each Tead, and in the other panels p-value is with respect to the equivalent values of the siControl. N = 3 in panels.

We previously showed that Tead4 plays an essential role in C2C12 cell differentiation [21]. To extend the study of Tead4 function, we isolated primary myoblasts from 3–4 week-old C57BL/6 mice and differentiated them *in vitro* for 6 days. Quantitative RT-PCR analysis showed that *Tead4* mRNA expression was strongly induced at days 3 and 6 during differentiation (Fig 1A). Similarly, expression of *Tead1* was also strongly induced, whereas *Tead2* expression did not show strong variation and *Tead3* was not significantly expressed in myoblasts. Immunoblots confirmed that Tead4 protein was strongly induced upon differentiation, whereas Tead1 protein was present in undifferentiated and differentiated cells (S1A Fig).

To address the function of Tead4, control and *Tead4* siRNAs were transfected 24 hours before the initiation of differentiation. Compared to control siRNA, si*Tead4* led to a potent reduction in *Tead4* expression, but did not affect *Myog* and *Myod1* levels (Fig 1B). Staining of the transfected cells for myosin heavy chain (hereafter Myh) expression showed that *Tead4* silencing did not impair myoblast differentiation as the *Tead4*-silenced cells formed long multinucleate myotubes (Fig 1C and 1D).

As *Tead1* was also present in differentiating PMs, we investigated potential redundancy amongst the Teads. We used siRNAs to silence *Tead1*, *Tead2* or *Tead4* and examined how this affected the expression of the other family members. *Tead1* expression was strongly down-regulated by si*Tead1*, less so by si*Tead2*, but not by si*Tead4* (Fig 1E and S1A Fig). Similarly, expression *of Tead4* mRNA was not affected by silencing of *Tead1* or *Tead2*, but Tead4 protein levels were increased in undifferentiated cells when *Tead1* was silenced. Expression of *Tead2* was reduced in the undifferentiated state upon silencing of either *Tead1* or *Tead2*, but its expression during differentiation was minimally affected. Thus, expression of each of the Teads was rather independent of the others.

As seen above, si*Tead4* silencing had little effect on differentiation (Fig 1F). Similarly, *Tead1* or *Tead2* silencing had little effect on differentiation (Fig 1F and 1G). Examination of gene expression showed nevertheless that *Myh1* and *Myh2* were reduced by each knockdown (Fig 1H). In contrast, expression of *Cav3*, a well-defined Tead4 target gene in C2C12 cells, and *Myh7* was strongly and selectively diminished in si*Tead4* cells indicating a specific requirement for Tead4 at these genes that cannot be compensated by expression of the other Teads.

**Fig 2:**
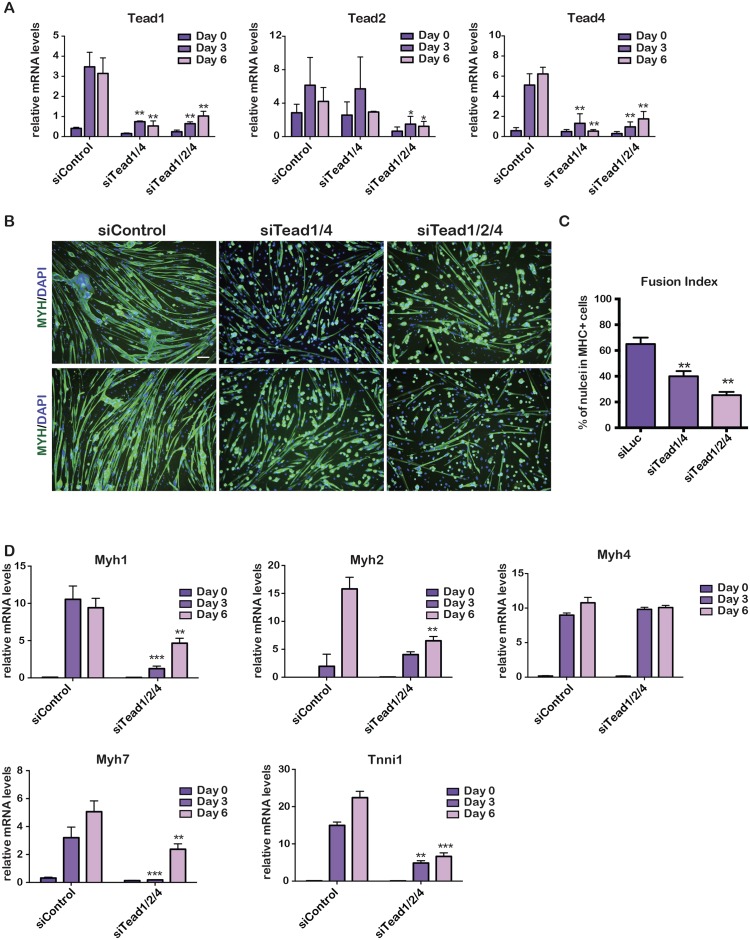
Redundant functions of Tead factors in PMs. **A**. Gene expression was quantified by RT-qPCR during PM differentiation after transfection with the indicated siRNAs. **B.** Fluorescence microscopy images after 6 days of differentiation of PMs transfected with the indicated siRNAs. Green channel shows staining with Myh antibody, and blue Dapi-stained nuclei. Scale bar 100 μm. **C.** Fusion index of siControl and siTead-transfected cells. **D.** Quantification of gene expression after transfection with the indicated siRNAs. In panels data was analysed by multiple t-tests. P-values as above. Fusion index was generated from counting around 500 nuclei in each condition from 3 biological replicates.

We performed combinatorial silencing of *Tead1* and *Tead4* or of all three expressed Teads. The expression of the corresponding *Tead* mRNAs was reduced in all cases (Fig 2A). In contrast to individual *Tead1* and *Tead4* knockdowns, their combinatorial knockdown had a potent effect on differentiation, with many cells expressing Myh, but no fusion and the prevalence of shorter myotubes (Fig 2B and 2C). Moreover, a larger number of cells failed to initiate Myh expression. Similar observations were made using the *Tead1*, *Tead2* and *Tead4* siRNA combination (Fig 2B). Strongly reduced *Myh1*, *Myh2*, *Myh7* and *Tnni1* expression was seen in the si*Tead1*/*Tead2*/*Tead4* cells (Fig 2D). Together, these observations showed that Teads play essential, but partially redundant functions in differentiating PMs.

### Specific and redundant roles of Tead family factors in C2C12 cell differentiation

The redundant roles for Tead factors in differentiating PMs contrast with the critical role for Tead4 in C2C12 cells. To investigate this more closely, we performed single and combinatorial siRNA Tead knockdowns in C2C12 cells.

**Fig 3:**
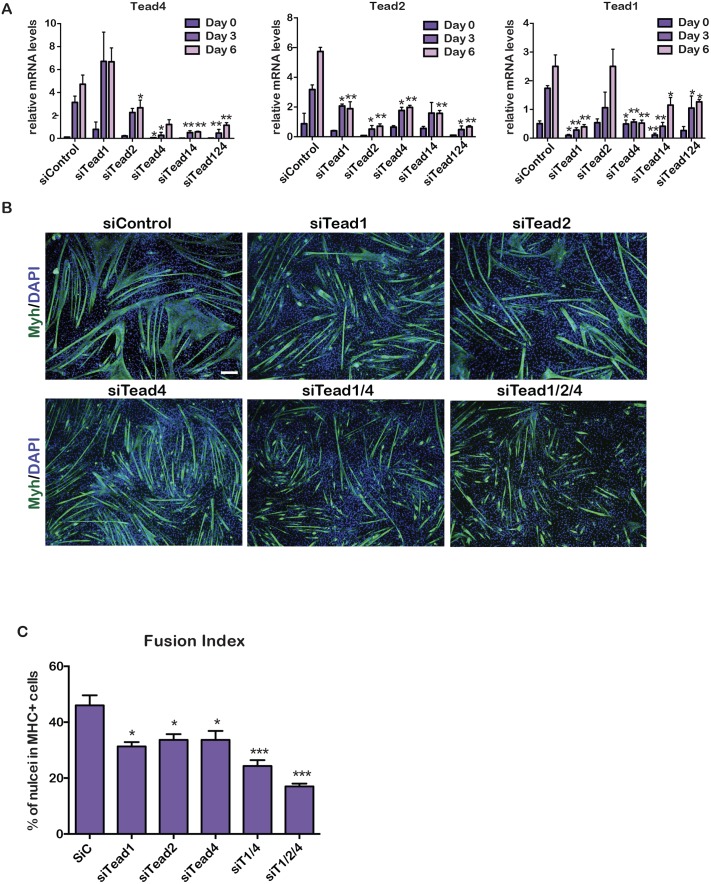
Tead factor function in C2C12 cells. **A**. Gene expression was quantified by RT-qPCR during C2C12 cell differentiation after transfection with the indicated siRNAs. **B.** Fluorescence microscopy images after 6 days of C2C12 cell differentiation following transfection of the indicated siRNAs. Green channel shows staining with Myh antibody. Scale bar 100 μm. **C.** Fusion index of siControl and following si*Tead* silencing. P-values as above. Fusion index was generated from counting around 1000 nuclei in each condition from 3 biological replicates.

As reported [21], *Tead4* was strongly induced during C2C12 cell differentiation (Fig 3A) and its induction was not diminished by *Tead*1 silencing, but was somewhat reduced by *Tead2* silencing. *Tead2* was also induced albeit less strongly than *Tead1* or *Tead4*, but its activation was strongly diminished by *Tead1* or *Tead4* silencing. *Tead1* mRNA was induced during differentiation, but this was strongly reduced by si*Tead4*. In all situations, transfection with siRNAs against individual Teads or combinations of Teads had the potent and expected effects on their own expression.

*Tead1* or *Tead4* silencing led to reduced myoblast fusion with the absence of longer and thicker fibres in favour of shorter less developed fibres (Fig 3B and 3C). A similar, but less pronounced, effect was seen upon *Tead2* silencing. Combinatorial *Tead1*/*Tead4* silencing led to more dramatic effects with fewer and shorter fibres, while upon silencing of all three Teads few elongated myotubes were observed (Fig 3B and 3C). These results revealed that normal expression of each Tead was essential for full differentiation and generation of long and thick fibres, and that Tead1 and Tead4 both strongly contributed to differentiation.

Western blot analyses showed increased Tead4 protein levels in differentiated cell extracts (S1B Fig). Tead1 on the other hand was decreased at day 6 in agreement with previous observations [22]. While Tead4 was increased in si*Tead1* cells, Tead1 was reduced in si*Tead4* cells. This highlights a difference with PMs where *Tead1* was strongly induced even in the absence of *Tead4* (S1A Fig), whereas in C2C12 cells *Tead4* is required for maximal *Tead1* expression.

Immunostaining showed Tead1 nuclear localisation in non-differentiated C2C12 cells, whereas Tead4 was present in both the nucleus and cytoplasm (S1C Fig). At day 6, Tead1 remained nuclear in cells that did not undergo differentiation, but was absent from differentiated myotube nuclei. In contrast, Tead4 expression was not detected in cells that did not undergo differentiation, but showed strong nuclear staining in myotubes. Strikingly, a comparison with PMs showed that Tead1 was strongly expressed in the nuclei of both myoblasts and myotubes, while Tead4 was both cytoplasmic and nuclear in myoblasts, but nuclear in myotubes (S1D Fig). This observation could account for the differential requirement for Tead1 and Tead4 in PMs and C2C12 cells. In PMs, both proteins were nuclear in myotubes and can thus partially compensate for each other, whereas in C2C12 myotubes, Tead1 was down-regulated by siTead4 and absent from the nucleus and thus unable to compensate for loss of Tead4.

### Selective Tead1 and Tead4 genomic occupancy

To understand how Tead1 and Tead4 regulate gene expression in C2C12 cells, we used ChIP-seq to profile their genomic occupancy. Chromatin was prepared before differentiation and after 6 days of differentiation and ChIP was performed with antibodies selective for either Tead4 or Tead1.

**Fig 4:**
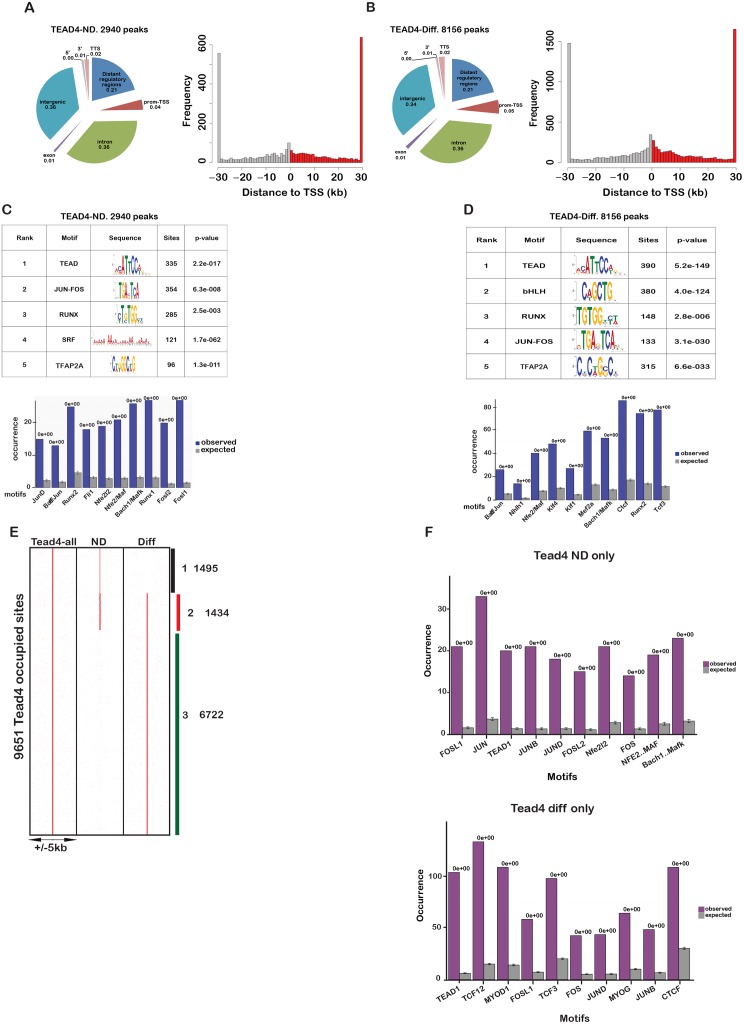
Tead4 genomic occupancy in C2C12 cells. **A-B.** Localisation of Tead4 occupied sites in non-differentiated and differentiated C2C12 cells relative to genomic annotations (left panels) and the TSS (right panels). **C-D.** Results of MEME analysis on the top 600 Tead4 occupied sites in non-differentiated and differentiated C2C12 cells. Lower panels indicate the frequency of occurrence of DNA binding motifs for the indicated transcription factors at Tead4 occupied sites in undifferentiated and differentiated C2C12 cells comparing the expected values with the observed values. **E.** Read density cluster map using a non-redundant list of all Tead4-occupied sites to compare occupancy in non-differentiated and differentiated cells. **F.** Analysis of enriched transcription factor binding motifs at Tead4 sites in differentiated and non-differentiated cells.

In undifferentiated cells, Tead4 occupied 2940 sites located mainly distant from the transcription start sites (TSS) (Fig 4A and S1 Dataset). In differentiated cells, more than 8100 sites were occupied, the majority of which were again located distant from the TSS (Fig 4B and S1 Dataset). Occupied sites in both undifferentiated and differentiated cells showed strong enrichment of the MCAT motif (Fig 4C and 4D). Other motifs co-occurred with the MCAT motif at higher than expected frequency. In undifferentiated cells, enrichment in motifs for the AP1 (Fos and Jun) family was observed along with Runx1 that cooperates with AP1 and Myod1 to drive myoblast proliferation during muscle regeneration [23] (Fig 4C). In differentiated cells, AP1 and Runx motifs were enriched, but additional motifs became prominent such as Ctcf, and Tcf3 and the E-Box (Fig 4D).

Comparison of sites in undifferentiated and differentiated cells indicated that occupancy of 1495 sites was lost during differentiation, whereas more than 6700 sites were gained and 1434 sites were occupied under both conditions (Fig 4E). For example, Tead4 constitutively occupied sites upstream of the *Ctgf* and *Ccnd1* genes, whereas occupancy of sites at the *Acta1* locus is seen only during differentiation (S2A Fig). Sites specifically occupied in undifferentiated and differentiated cells showed enrichment in AP1 motifs in the undifferentiated state, but enrichment of Myod1, Myog, Tcf3 and Tcf12 that cooperates with Myod1 to promote myogenic differentiation [24] in differentiated cells (Fig 4F).

To assess co-localisation between Tead4 and transcription factors whose motifs were enriched at its binding sites, we compared the Tead4 profiles with those of Jun and Srf in C2C12 cells and Runx1 in primary myoblasts [25] [26]. Around 35% of sites bound by Tead4 in undifferentiated and differentiated cells overlapped with those bound by Jun (S2B Fig). In contrast, there was little overlap with the sites preferentially bound in the differentiated state, where the frequency of co-occurring AP1 motifs was also reduced. Similarly, a strong binding overlap between Tead4, Runx1 and Srf was seen involving sites occupied in undifferentiated and differentiated cells.

A similar analysis of Tead1 identified 1400 bound sites in undifferentiated cells, enriched in MCAT, AP1 and Runx motifs (S3A and S3B Fig). Nevertheless, in agreement with its absence from the differentiated cell nucleus, Tead1 occupancy was strongly reduced in the differentiated state with only 274 detected sites (S3C Fig). Even at sites occupied in both conditions, Tead1 occupancy was reduced in the differentiated state (S3D Fig). Thus, transition to the differentiated state involved a switch from Tead1 and Tead4 occupancy to predominantly Tead4 occupancy.

In the undifferentiated state, Tead1 and Tead4 co-occupy more than 900 sites (S3E Fig). A set of sites showed preferential occupancy by Tead1, but only few sites showed exclusive Tead1 occupancy. Thus despite the fact that these two proteins bind identical sequences and that Tead1 occupancy was globally lower than Tead4, a set of sites was preferentially occupied by Tead1.

As Tead4 regulated *Tead1* expression during differentiation, we examined Tead4 occupancy at the *Tead1* locus. Two constitutive Tead1/Tead4 occupied sites were observed, one upstream of the promoter of the longest isoform and a second upstream of an alternative promoter for a shorter isoform (S4A Fig). During differentiation Tead1 occupancy diminished, but Tead4 occupancy was maintained suggesting that Tead4 directly regulates *Tead1*. Integration of Tead1/4 ChIP-seq data with public data on histone modifications in undifferentiated and differentiated C2C12 cells revealed the presence of H3K27ac, a mark of active promoters and enhancers, at the *Tead1* promoter in undifferentiated cells overlapping with the Tead1/4 occupied sites. Interestingly, upon differentiation, H3K27ac increased at the Tead1/4 occupied sites and new regions marked by H3K27ac appeared upstream of and overlapping with the alternative promoter. Moreover, integration with public ChIP-seq data indicated Myod1 and Myog [27] binding at the H3K27ac-enriched regions. These observations suggest that Tead4 cooperates with Myog and Myod1 to activate *Tead1* expression during differentiation via constitutive and inducible enhancer elements, in agreement with *Tead1* down-regulation in si*Tead4* knockdown C2C12 cells (Fig 3A).

At the *Tead4* locus, Tead4 occupied a H3K27ac-marked site immediately upstream of its own promoter whose occupancy increased upon differentiation (S4B Fig). In contrast, almost no Tead1 occupancy was seen. Upon differentiation, regions in the *Tead4* gene body acquired H3K27ac, several which coincided with binding of Myod1 and Myog. This suggests that Tead4 positively regulates its own expression together with these factors that bind to differentiation-induced enhancer elements downstream of the Tead4 TSS.

The above data show that at the *Tead1* and *Tead4* loci, enhancers binding Myog became activated during differentiation perhaps driving their expression. We tested this by performing si*Myog* silencing in C2C12 cells and in PMs. In both cell types, si*Myog* strongly inhibited differentiation (S5A Fig). In C2C12 cells, *Tead4* expression was reduced upon *Myog* silencing, while that of Tead1 was unaffected, and *Ccnd1* expression was increased (S5B Fig). Expression of *Mef2c* bound by both Myog and Tead4 (S5C Fig) was also strongly repressed. Hence, Myog is required for Tead4, but not Tead1, expression and differentiation.

**Fig 5:**
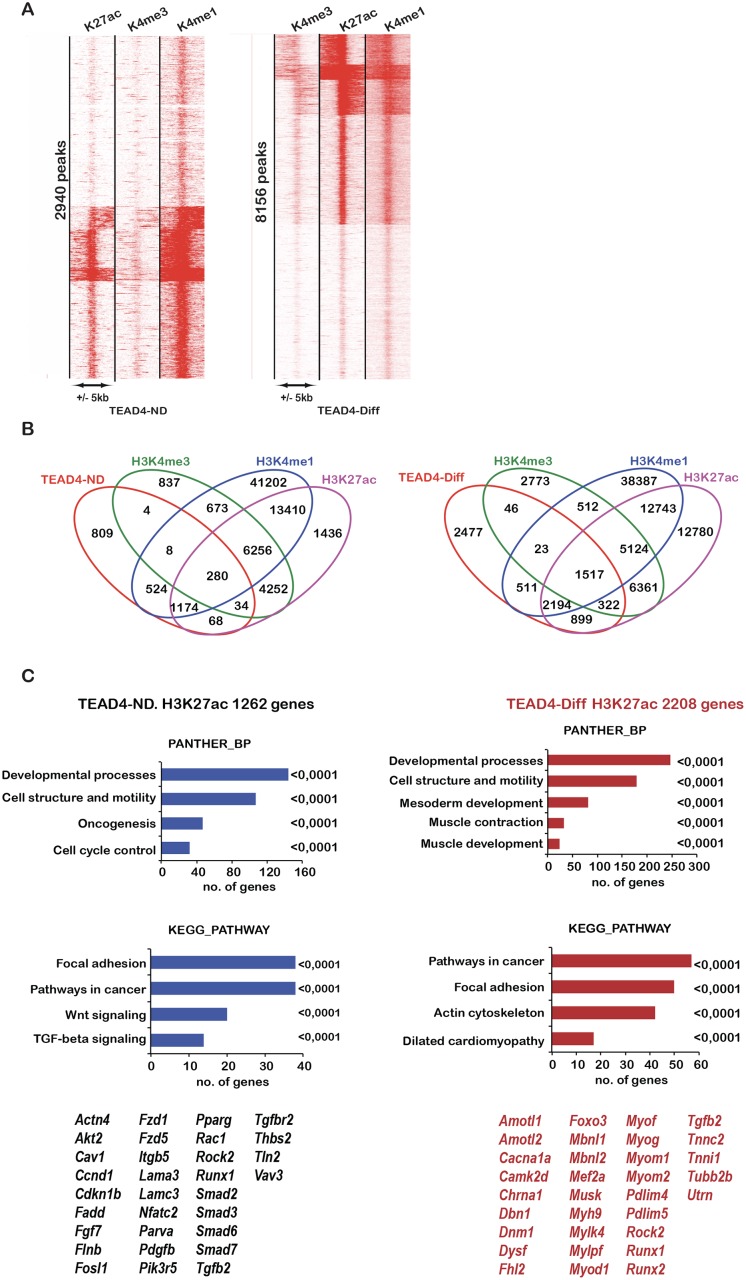
Integration of Tead4 genomic occupancy with chromatin modifications during C2C12 cell differentiation. **A.** Read density cluster map showing chromatin modifications at Tead4-occupied sites in non-differentiated and differentiated cells. **B.** Venn diagrams illustrating the overlap of chromatin modifications with Tead4 genomic occupancy. **C.** Identification and ontology analysis of genes associated with Tead4 sites at active H3K27ac marked regulatory elements.

In a global analysis, we clustered Tead4-occupied sites in undifferentiated and differentiated cells with H3K4me3 a mark of active promoters, H3K4me1, a mark of active and poised enhancers as well as H3K27ac. As few Tead4 sites localised at the TSS, only a limited overlap (280 of 2940) with H3K4me3 was observed (Fig 5A and 5B). In contrast, 1698 Tead4 sites in undifferentiated cells showed strong association with H3K4me1 and/or H3K27ac defining a set of sites at active and poised enhancer elements. A similar situation was seen in differentiated cells where almost half were marked by H3K4me1 and H3K27ac and up to 1500 sites associated with H3K4me3 (Fig 5A and 5B). Tead4 therefore occupied a set of functional regulatory elements in both undifferentiated and differentiated cells. A similar situation was seen for Tead1 in undifferentiated cells (S6A and S6B Fig). Due to their low number, we did not analyse Tead1 sites in differentiated cells.

To define the regulatory potential of Tead4, we identified the genes closest to the Tead4-occupied sites associated with active chromatin marks. In undifferentiated cells, 1262 genes enriched in the ontology terms cell structure and motility, developmental processes, oncogenesis and cell cycle control were annotated (Fig 5C). Interestingly, KEGG pathway analysis revealed that Tead4 (and Tead1, S6C Fig) occupied sites associated with critical components of the Tgfβ (*Smad2*, 3 6 and 7 as well as *Tgfb2*) and Wnt-signalling (*Fzd1*, *Fzd5*, *Tcf7l2*) pathways (Fig 5C). In addition, several genes of the Hippo pathway such as *Amotl1*, *Amotl2* and *Lats2* were also associated with Tead1/4 occupied sites. In differentiated cells, more than 2000 genes enriched in terms associated with developmental processes, muscle differentiation and contraction were annotated including the important regulatory genes *Myod1*, *Myog* and *Mef2a* as well as numerous structural genes of the muscle fibre. At many sites, Tead4 binding and H3K27ac was either enriched or acquired *de novo* at these genes during differentiation (S6D Fig).

We analysed global co-localisation of Tead4 with Myod1- and Myog-occupied sites. In differentiated cells, more than 2000 Tead4 sites were co-occupied by all three factors (S7A Fig and S2 Dataset). As Tead1 occupied sites essentially only in non-differentiated cells, a comparison with Myod1 and Myog-occupied sites in differentiated cells revealed only a limited overlap of around 50 sites (S7B Fig). The Tead4-Myod1-Myog-occupied sites showed enrichment not only in the recognition motifs for these factors, but also for Tcf3, Tcf12, Runx, and Klf5, whereas the AP1 family sites were less represented than in the overall Tead4 profile (S7C Fig). We compared Tead1/4 occupancy with that of Mef2a, another myogenic factor for which a public data set is available in undifferentiated C2C12 cells [28] and identified a set of sites co-occupied with Tead1 and Tead4 (S7E and S7F Fig).

This analysis identified Tead4 sites closely associated with Myod1/Myog. Nevertheless, as shown above at the *Tead4* and *Mef2c* loci, Tead4 may cooperate with Myod1/Myog to activate these genes despite more distant localization of the binding sites. We therefore defined genes associated with Tead4-occupied sites and compared them with genes associated with Myog/Myod1 sites to identify those potentially regulated by these factors despite binding more distantly spaced promoter and/or enhancer elements. A large majority of Tead4 associated genes was associated with Myog/Myod1 whose potential target genes also showed a strong overlap (S7D Fig)

### Tead1/4-regulated gene expression in differentiating C2C12 cells

**Fig 6:**
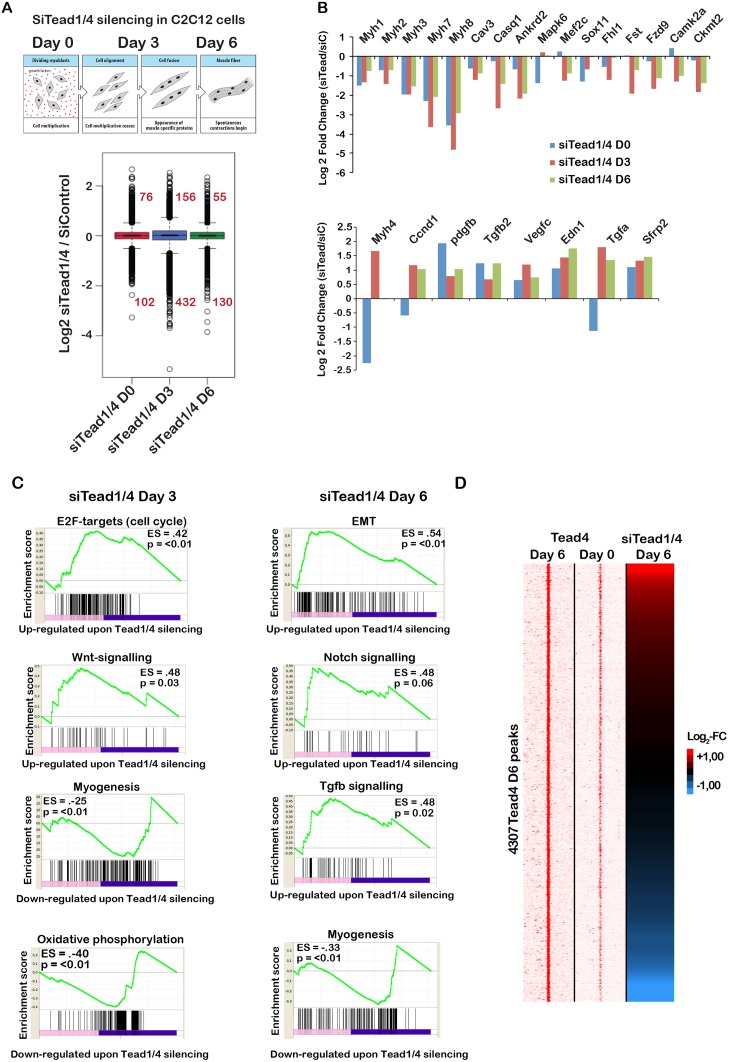
Tead-regulated genes in C2C12 cells. **A.** Schematic outline of the experimental design and box plots showing gene expression changes during differentiation of cells transfected with *siTead1/4*. **B.** Examples of genes deregulated in *siTead1/4* cells compared to siControl. **C.** GSEA analyses of Tead1/4 regulated genes. The most significant categories in the up- and down-regulated genes sets are shown. **D.** Tead4 sites preferentially occupied in differentiated cells and associated with expressed genes are depicted in a read density map alongside a heat hap of gene expression following si*Tead1/4* knockdown.

We used RNA-seq to investigate gene expression in differentiating PMs and C2C12 cells and how simultaneous Tead1 and Tead4 silencing affected these regulatory programs to impair differentiation. SiRNAs were transfected and RNA prepared after 24 hours (day 0) before cells were moved to differentiation media and RNAs prepared 3 and 6 days later (Fig 6A). Changes in expression in si*Tead1/4* compared to the siControl were quantified to identify genes showing a greater than Log2 fold change of 1 with adjusted p value <0,05.

In control C2C12 cells, 3137 genes were induced at day 3 and day 6 with respect to day 0, while in PMs 3626 genes were induced of which 1845 genes were commonly induced in both cell types (S8A Fig). Commonly regulated genes were associated with muscle differentiation. Similarly, 2375 genes were repressed during C2C12 cell differentiation and 2799 genes repressed in PMs with 1495 common to both cell types (S8B Fig). Commonly repressed genes were associated with cell cycle, consistent with proliferation arrest during differentiation. Thus, similar but not identical, gene expression programs were activated and repressed during the differentiation of these two cell types.

Analysis of the 5512 genes regulated during differentiation of siControl C2C12 cells identified genes with different expression profiles that could be summarised in 6 clusters (S9A Fig). Genes in clusters 1, 3 and 4 were down-regulated with different kinetics, while those in clusters 2 and 5 were up-regulated with different kinetics, and those in cluster 6 were transiently induced at day 3. Further analyses showed that myogenic genes were amongst the most significantly up-regulated at days 3 and 6, whereas cell cycle progression genes were strongly repressed (S9B Fig). In addition, adipogenesis genes were also induced along with a metabolic switch involving increased expression of genes involved in oxidative phosphorylation.

Following *siTead1/4* silencing, up and down-regulated genes were seen at day 0. The number of de-regulated genes increased at day 3 and diminished by day 6 (Fig 6A). In total, 249 genes were up-regulated by *siTead1/4* silencing between day 0–6, while 549 were repressed (Fig 6A and 6B and S3 Dataset). Analysis up-regulated genes at days 3 and 6 indicated strong enrichment in cell cycle, Notch, Wnt and Tgfβ signalling and epithelial to mesenchymal transition (Fig 6C). In contrast, genes involved in myogenesis and oxidative phosphorylation were repressed. Hence, Tead1/4 contribute to activation of the myogenic differentiation program, but they also directly or indirectly repress growth promoting pathways leading to defective cell cycle arrest.

To identify genes directly regulated by Tead4, we determined those within 50 kb of Tead4 binding sites enriched specifically in differentiated cells. Around 4300 Tead4 occupied sites were associated with 4100 potential target genes showing expression in the RNA-seq data (Fig 6D). A set of 172 down-regulated genes enriched in muscle differentiation functions was associated with Tead4 binding sites in differentiated cells (Fig 6D). Similarly, a set of 107 up-regulated genes was associated with sites preferentially bound in the differentiated state. These genes were enriched in the cell cycle, Notch and Wnt signalling identified by the GSEA analyses. Hence, binding of Tead4 to these repressed genes during differentiation provided further evidence that Tead4 contributed to their repression.

### Tead1/4-regulated gene expression in differentiating primary myoblasts

A similar analysis of differentiating PMs clustered gene expression (S10A Fig) in 6 kinetic classes and showed that differentiation was characterised by activation of genes involved in myogenesis, oxidative phosphorylation and adipogenesis, while cell cycle genes were repressed (S10B Fig).

**Fig 7:**
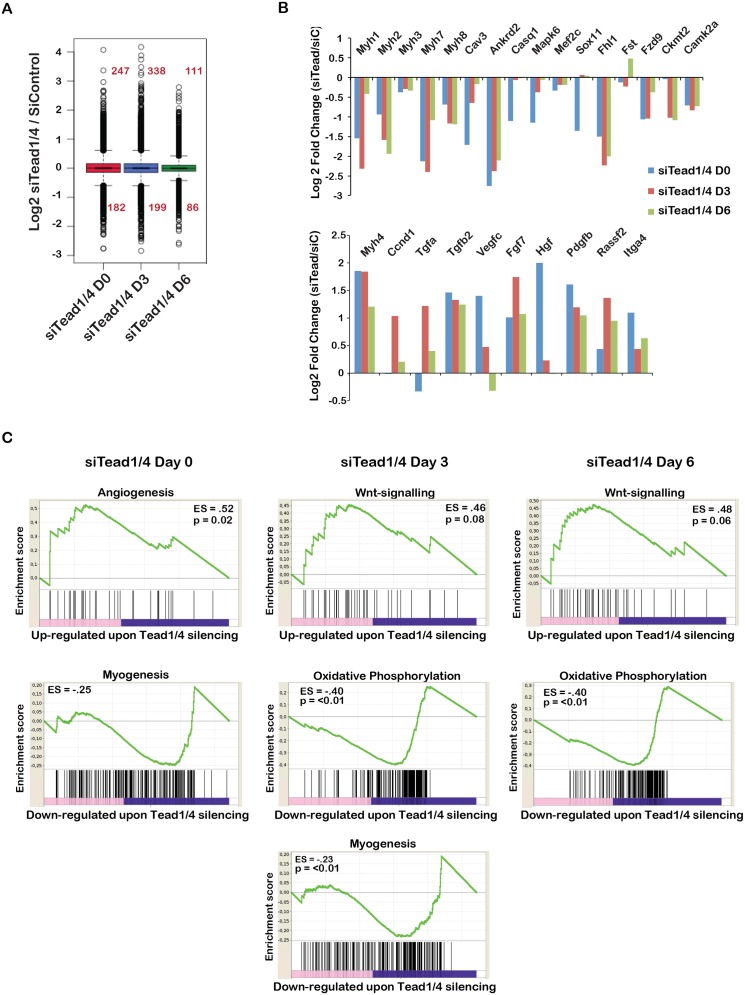
Tead-regulated genes in PMs. **A.** Box plots showing gene expression changes during differentiation of PMs transfected with *siTead1/4*. **B.** Examples of genes deregulated in *siTead1/4* cells compared to siControl. **C.** GSEA analyses of Tead1/4 regulated genes. The most significant categories in the up- and down-regulated genes sets are shown.

Following si*Tead1/4* silencing, deregulated genes were observed at day 0, increased at day 3 and then diminished at day 6 (Fig 7A). In total, 563 genes were up-regulated between day 0–6, while 377 were repressed (Fig 7A and 7B and S4 Dataset). Down-regulated genes were associated with myogenesis, and oxidative phosphorylation, the hallmarks of differentiation, whereas up-regulated genes were enriched in angiogenesis and as seen in C2C12 cells in Wnt signalling (Fig 7C). In PMs, up-regulation of cell cycle genes was observed, but the values were less significant reflecting the reduced proliferative capacity of PMs compared to C2C12 cells. Thus, Tead factors were essential to activate genes involved in PM differentiation, but also to repress Wnt signalling and signalling pathways like Tgfβ inhibiting PM differentiation.

We compared genes de-regulated by si*Tead1/4* silencing in PMs and C2C12 cells. As the kinetics of their activation of repression may differ, we compared non-redundant lists of all genes deregulated between days 0–6 in each cell type. A set of 119 genes strongly enriched in muscle differentiation functions were commonly down-regulated (S11A Fig). Strikingly however, a large set of 430 genes, again strongly enriched in muscle differentiation functions, was specifically down-regulated in C2C12 cells (S5 Dataset). A smaller set of 258 genes was specifically down-regulated in PMs, but showed low enrichment in more diverse functions. Only 65 genes involved in signalling and proliferation were commonly up-regulated. However, a large set of almost 500 genes was specifically up-regulated in PMs (S11B Fig). Remarkably, these genes showed enrichment in nervous system development and other neurogenesis functions (S5 Dataset). Tead1/4 knockdown appeared to modify PM cell identity leading to the expression of neurogenesis genes, not normally expressed during PM differentiation. These results showed that Tead1/4 silencing had distinct effects on gene expression in PMs and C2C12 cells.

### Genome occupancy by Tead4 in muscle *in vivo*

**Fig 8:**
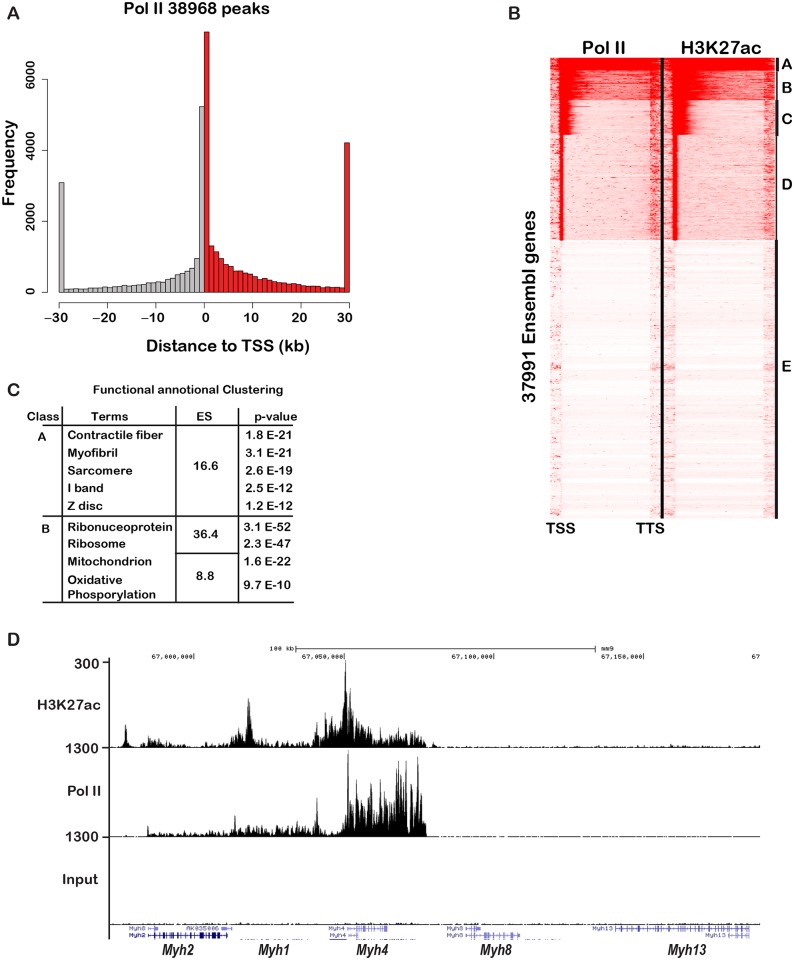
Highly transcribed muscle cell identity genes. **A.** Localisation of Pol II peaks in muscle relative to the TSS. **B.** Read density analyses of Pol II and H3K27ac at Ensembl annotated genes distinguishing those with high transcribing Pol II density classes A and B from those with lower or no transcribing Pol II, classes C-E. **C.** Results of functional enrichment ontology analyses of the genes in classes A and B showing the enriched terms, the enrichment score (ES) and the p-values. **D.** UCSC genome browser view showing Pol II and H3K27ac profiles at the *Myh4* locus.

We next addressed genome occupancy by Tead4 in mouse muscle *in vivo*. We developed a protocol to prepare chromatin from dissected hind-limb muscle (see Materials and methods) and performed ChIP-seq for Tead4, H3K27ac and RNA Polymerase II (Pol II). We analysed the Pol II ChIP-seq to determine whether the signal obtained reflected mainly Pol II occupancy in muscle or in contaminating non-muscle cells. More than 38000 Pol II peaks were identified most of which localised at the TSS (Fig 8A). Transcribed genes can show high levels of promoter paused Pol II and low levels in the gene body or low pausing, but abundant elongating Pol II [29]. The second class often corresponds to tissue identity genes controlled by so-called “super enhancers” [30, 31]. Analyses of the Pol II ChIP-seq data identified around 1000 genes with high levels of transcribing Pol II (class A in Fig 8B), a second class (B) also with high Pol II in the transcribed regions and larger groups of genes (C and D) with high Pol II at the promoter, but lower levels in the gene body. Class A genes also showed high levels of H3K27ac throughout the gene body typical of what has been described at cell identify genes (Fig 8B). Class A genes associated with high Pol II and H3K27ac were enriched in terms associated with muscle fibre (Fig 8C). For example, the locus comprising *Myh2*, 1, 4, 8 and 13 showed high Pol II density specifically over the *Myh4* gene with much lower densities over the *Myh1* and *Myh2* genes, but no transcription of other myosin genes at this locus (Fig 8D). These loci also showed extensive H3K27ac surrounding and throughout the gene body. The Pol II and H3K27ac ChIP-seq therefore identified a set of highly transcribed muscle identity genes confirming the signal comes predominantly from muscle cells.

**Fig 9:**
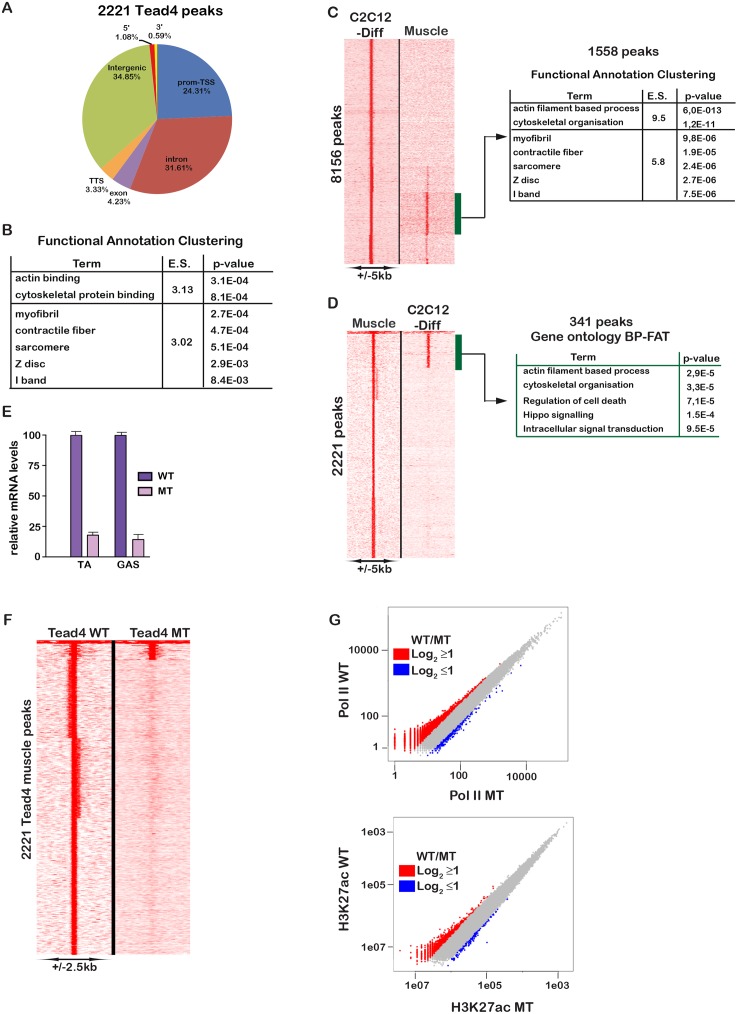
Tead4 occupancy in mature muscle *in vivo*. **A.** Localisation of Tead4 binding sites relative to the genome. **B.** Results of functional enrichment ontology analyses of the genes associated with Tead4 bound sites in muscle showing the enriched terms, the enrichment score (ES) and the p-values. **C-D.** Comparison of Tead4 binding in differentiated C2C12 cells and in muscle *in vivo*. Panel C uses coordinates of C2C12 Tead4 sites as reference and panel D, the mature muscle Tead4 sites. The results of functional enrichment ontology analyses of the genes associated with the shared sites showing the enriched terms, the enrichment score (ES) and the p-values. **E.** RT-qPCR of *Tead4* expression in tibialis anterior and gastrocnemius muscles from *Hsa*::Cre-ER^T2^::*Tead4*^lox/lox^ mice with (MT) or without (WT) Tamoxifen injection. **F**. Read density maps of Tead4 occupancy in WT and MT muscle. **G**. Global peak occupancy profiles of Pol II and H3K27ac in wild-type and Tead4-mutant muscle.

Of the 2220 identified Tead4 sites, 686 were associated with active promoters marked by Pol II and H3K27ac and enriched in muscle specific functions (Fig 9A and S12A Fig). Genes associated with Tead4-bound sites showed enrichment in terms associated with muscle structural proteins (Fig 9B). Aligning the muscle Tead4 ChIP-seq to the coordinates of the differentiated C2C12 cell peaks revealed 1558 sites with significant signal (Fig 9C). Genes associated with these shared sites were enriched in muscle structural proteins. In the converse comparison using the top 2200 Tead4-bound sites in muscle as reference, 341 common peaks were identified (Fig 9D). These comparisons revealed Tead4 sites in muscle that were not called amongst the 2200 high confidence sites, but although showing lower occupancy in muscle were shared with differentiated C2C12 cells. Tead4 therefore bound a distinct repertoire of sites in C2C12 cells and muscle and sites with high occupancy in muscle did not necessarily show high occupancy in C2C12 cells and *vice-versa*.

We next performed ChIP-seq from muscle of mice in which Tead4 was specifically inactivated in fibres using the *Hsa*::Cre-ER^T2^ driver [32]. Mice with floxed *Tead4* alleles were crossed to generate *Hsa*::Cre-ER^T2^::*Tead4*^lox/lox^ animals. These mice were injected at 6–7 weeks with tamoxifen for 4 consecutive days and 3 weeks after injection *Tead4* expression was strongly reduced in the tibialis anterior and gastrocnemius muscles showing efficient recombination (Fig 9E). We performed Pol II, H3K27ac and Tead4 ChIP-seq from these *Tead4*^musc-/-^ animals. Aside a small number of sites with signal in *Tead4*^musc-/-^ animals, Tead4 binding was lost (Fig 9F) indicating that observed signal came almost exclusively from sites bound in muscle. Comparison of Pol II and H3K27ac profiles in wild-type and *Tead4*^musc-/-^ muscle (Fig 9G) showed only minor changes in low intensity signals and hence that Tead4 loss did not affect global Pol II or H3K27ac distribution.

For example, at the *Acta1* locus Tead4 occupancy was lost in mutant muscle, whereas no change in Pol II and H3K27ac profiles was observed (S13 Fig). Similarly, Tead4 occupied 3 sites at the *Amolt2* locus in both C2C12 cells and wild-type muscle including an upstream enhancer site marked by H3K27ac (S14 Fig). The shared sites co-localised with those occupied by Myod1 and Myog in C2C12 cells. In mutant muscle, Tead4 occupancy was lost, but no change for Pol II and H3K27ac was seen.

In agreement with the unaltered Pol II and H3K27ac profiles, little change in expression of potential Tead4 target genes was seen in mutant muscle, only minor reductions in *Myh2* and *Myl2* expression were observed (S15A and S15B Fig). Moreover, *Tead4*^musc-/-^ animals did not show any marked phenotype in terms of muscle fibre size, muscle mass and grip strength (S15C–S15E Fig). One potential explanation is redundancy with Tead1. To investigate this possibility, we performed Tead1 ChIP-seq in muscle. Of the 358 sites identified, 188 were shared with Tead4 (S12B Fig). Genes associated with Tead1-occupied sites were however enriched in muscle function. For example, prominent Tead1 and Tead4 occupancy was observed at the *Acta1* and *Amotl2* loci (S13 and S14 Figs). Thus, redundancy with Tead1 may in part account for the lack of phenotype seen upon Tead4 inactivation. Alternatively, differentiating C2C12 cells and PMs represent a very different physiological state from mature differentiated fibres. A more comparable situation is muscle fibre regeneration. We therefore investigated the role of Tead4 in muscle fibre regeneration *in vivo*.

**Fig 10:**
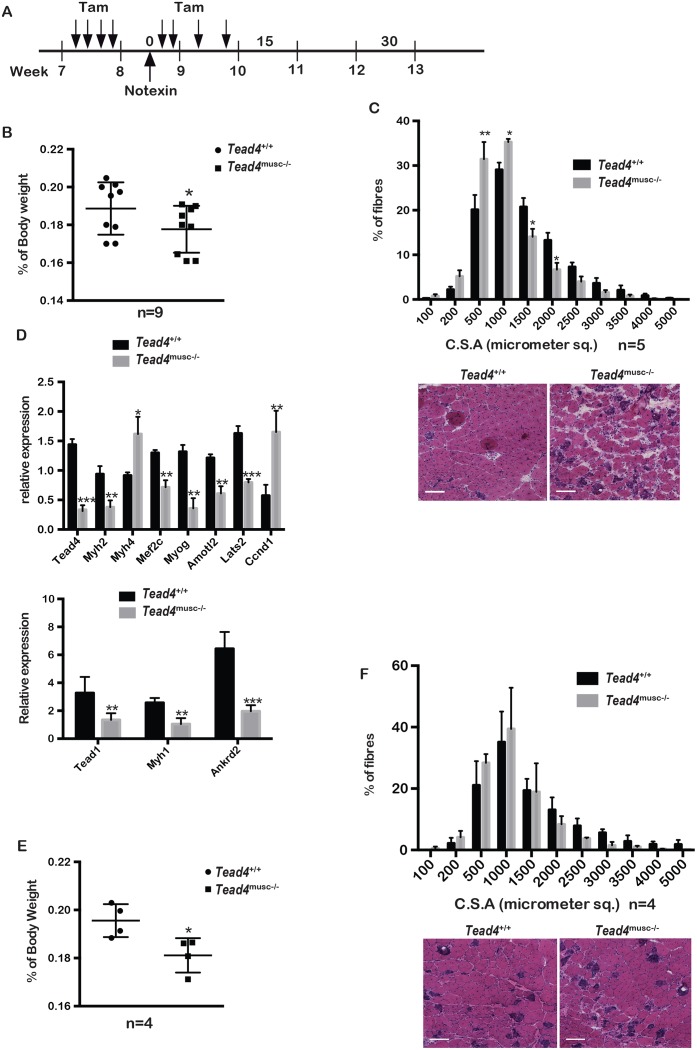
Tead4 is required for muscle regeneration *in vivo*. **A**. Outline of experimental protocol showing days of Tam and Notexin injection. **B**. Tibialis anterior mass of the indicated animals 15 days after notexin injection. Two-tailed t-test, p value< 0,05 on a total of 9 animals of each genotype from 2 cohorts. **C**. Tibialis anterior cross-section area of the indicated animals 15 days after notexin injection. Two-way anova, p-value ** <0,001, p-value * <0,01, N = 5. The lower panel shows a representative HE stained section, scale bar 100 μm. **D**. RT-qPCR of the indicated genes 15 days after notexin injection. T-test; p-value *** <0,0001, p-value ** <0,01, p-value * <0,05. N = 5. **E-F**. Tibialis anterior mass and cross-section areas of the indicated animals 30 days after notexin injection. N = 4.

We employed a protocol similar to that previously used to demonstrate the role of SRF in regeneration using the *Hsa*::Cre-ER^T2^ driver ([33] and see Fig 10A). Muscle degeneration in *Hsa*::Cre-ER^T2^::*Tead4*^lox/lox^ and *Hsa*::Cre-ER^T2^::*Tead4*^+/+^ animals was induced by notexin injection and Tead4 was inactivated in regenerating fibres by regular subsequent Tam injection (Fig 10A). At 15 days after notexin injection, tibialis anterior mass was significantly lower in the *Tead4*^musc-/-^ compared to the *Tead4*^+/+^ animals (Fig 10B). Similarly, fibre cross-section area was significantly altered with more small fibres and less large fibres in *Tead4*^musc-/-^ (Fig 10C). Expression of several Tead4 target genes such as *Myh1* and *Myh2*, *Ankrd2*, *Lats2* and *Amotl2* were all significantly reduced (Fig 10D). Moreover, *Tead1* and *Myog* expression were also reduced, whereas *Ccnd1* expression was increased. Thus, the gene expression changes induced by Tead4 inactivation during notexin-induced regeneration were similar but not identical to those seen following si*Tead4* in PMs.

At 30 days after notexin injection, tibialis anterior mass remained somewhat reduced in the mutant animals, but fibre cross-section area was comparable to that in control animals (Fig 10E and 10F). Thus, Tead4 inactivation delayed the normal regeneration process.

## Discussion

### Tead factors are essential for myogenic differentiation *in vitro*

Here we show the essential role of Tead factors in PM differentiation. While silencing of each individual Tead had little discernible effect at the cellular level, Tead4 silencing specifically affected expression of its direct targets *Myh7* and *Cav3*. Nevertheless, combinatorial *Tead1/4* or *Tead1/2/4* silencing strongly impaired PM differentiation with fewer cells initiating Myh expression and shorter myotubes. Functional redundancy may be explained by the persistent expression and nuclear localisation of *Tead1* during differentiation of si*Tead4* PMs and *vice-versa*.

In contrast, si*Tead4* silencing impaired C2C12 cell differentiation with formation of shorter myotubes. Individual *siTead1* or *siTead2* silencing also impaired differentiation, revealing differences in Tead contributions in PM and C2C12 cells. In C2C12 cells, *Tead4* silencing diminished *Tead1* and *Tead2* expression. Indeed, Tead4 occupied *Tead1* regulatory sequences to directly regulate its expression. In addition, while Tead1 and Tead4 were nuclear in differentiated PMs, Tead1 was absent from the differentiated C2C12 cell nuclei and therefore could not compensate Tead4 silencing. C2C12 cell differentiation is however impaired by si*Tead1* showing that it contributed to early events in this process. Differential contribution of Teads in the two cell types can thus be explained by differences in their regulation and intra-cellular localisation.

Immunostaining detected Tead1 uniquely in the nucleus of non-differentiated C2C12 cells, whereas Tead4 expression was lower and distributed in both nucleus and cytoplasm. However, ChIP-seq showed higher genomic occupancy of Tead4 than Tead1 suggesting its preferentially recruitment to the non-differentiated cell genome. While it is possible that the ChIP-efficiency of the Tead4 antibody is higher than the Tead1 antibody, a set of sites showed preferential occupancy by Tead1 rather suggesting the overall lower binding of Tead1 cannot simply be explained by lower ChIP efficiency. Indeed, it has previously been reported that the Vgll2 cofactor induced during C2C12 cell differentiation inhibits Tead1, but not Tead4 DNA binding [22]. Hence, it is possible that during differentiation Vgll2 acts to selectively inhibit Tead1 genomic binding leading either to its export from the nucleus and/or its reduced expression.

In our previous study [21], we performed ChIP in cells constitutively overexpressing tagged Tead4. Despite constitutive Tead4 overexpression, we identified sites occupied only during differentiation consistent with their acquisition of H3K4me3 or H3K27ac. Others, exemplified by a site upstream of the Myog locus (see S6D Fig), were occupied by exogenous, but not endogenous Tead4 in proliferating C2C12 cells. Thus, while Tead4 occupies many sites in undifferentiated C2C12 cells, there exists a subset of sites occupied only during differentiation irrespective of Tead4 expression levels, whereas others can be occupied in the undifferentiated state upon increased Tead4 expression. Tead4 genome occupancy is therefore not only regulated by its expression level, but also by changes in chromatin state during differentiation.

Integration of Tead4 ChIP-seq data with that of chromatin modifications showed strong association of Tead4 occupied sites with active H3K27ac-marked regulatory elements in both undifferentiated and differentiated cells. Moreover, many sites showed Tead4/Myog/Myod1 co-occupancy. These observations reinforce the idea that Tead4 in particular and Teads in general may cooperate with Myod1 and Myog to regulate gene expression during differentiation.

Myod1 orchestrates the activation of a compendium of muscle enhancer elements [25] [34]. The DNA sequences at these enhancers were enriched for the AP1 and Runx families, but not for the MCAT motif. Tead4-occupied sites in non-differentiated cells were enriched in AP1 and Runx motifs suggesting these factors collaborate to drive proliferation. Recently, sites occupied by Tead factors driving motility in cancer cells were identified and showed not only enrichment in AP1 motifs, but also many of the motifs that we found enriched at Tead4 sites in C2C12 cells [35]. In contrast, Tead4 sites preferentially occupied in differentiated cells showed little overlap with Jun, but better co-localisation with Runx and were enriched for E-boxes for Myod1/Myog consistent with their observed co-localisation. Nevertheless, although many Tead4 occupied sites are co-occupied by Myod1/Myog, these sites constitute only a smaller subset of a larger collection of Myod1/Myog sites explaining why the MCAT motif was not detected in the analyses of Blum et al., [25].

RNA-seq showed that Tead1/4 drive distinct but overlapping gene expression programs in the two cell types. This partly reflects the different gene expression programs of PMs and C2C12 cells, but also suggests that Tead4 may occupy a different, but overlapping set of sites in these two cell types. Interestingly, a large number of muscle function genes are specifically down-regulated in C2C12 cells by Tead1/4 silencing. This may reflect the additional contribution of Mef2c that was down-regulated in C2C12 cells, but not PMs. More striking is the specific up-regulation of neuron-expressed genes in PMs suggesting that Tead1/4 silencing leads to altered cell identity. Nevertheless, many genes critical for fibre formation, contraction and neuromuscular junction are down-regulated by si*Tead1/4* in both cell types. Diminished expression of these genes contributes to the impaired differentiation observed.

We previously suggested that in addition to activating muscle differentiation genes, Tead4 may also repress genes such as *Ccnd1* and *Ctgf* that drive cell proliferation and whose expression is reduced upon differentiation [21]. While Tead4 binds genes like *Ccnd1* before and during differentiation, we observed here sites preferentially bound in differentiated cells and associated with cell cycle such as *E2f8* or *Chek2*, or Wnt, Tgfβ and Notch signalling genes. These genes are normally repressed during differentiation, but were up-regulated after si*Tead1/4* silencing. Proper regulation of Wnt, Notch and Tgfβ signalling is essential for normal myogenic differentiation [36] [37] [38] and their mis-regulation impairs myogenesis and can lead to fibrosis [39]. For example, si*Tead1/4* silencing up-regulated its target genes *Notch3*, and to a lesser extent *Notch1*, and *Dll1* ligand, accompanied by up-regulation of the Notch mediators *Hey1* and *Hey2* shown to inhibit myogenesis [40]. On the other hand, si*Tead1/4* silencing up-regulated its target gene *Nkd1*, an antagonist of Wnt signalling [41] [42] that is normally required to promote differentiation. Thus, Tead4 plays a dual role during differentiation, not only activating the myogenic program, but also repressing cell cycle and signalling genes.

Some discrepancies remain with our previous observations using sh*Tead4* silencing in C2C12 cells. For example, sh*Tead4* silencing strongly inhibited *Myog* expression, while this was not seen upon si*Tead4* silencing. This may reflect a fundamental difference in the two approaches. In the shRNA experiments, C2C12 myoblasts were infected, selected and Tead4 expression was silenced for up to 10 days before differentiation was initiated. As Tead4 occupies more than 2800 binding sites in proliferating myoblasts, it is likely that diminished Tead4 levels for several days prior to differentiation can affect activation of genes that are rapidly induced after differentiation. Extensive Tead4 genome occupancy in proliferating C2C12 cells may therefore play a critical role in establishing the proper chromatin state permissive for activation of genes during differentiation.

### Tead4 is essential for normal muscle regeneration

Performing ChIP-seq directly from mature muscle fibres identified a set of a highly transcribed and H3K27ac-marked muscle cell identity genes and Tead4 and Tead1 bound sites. Tead1 and Tead4 occupied an overlapping set of sites that partially overlapped with those in C2C12 cells. Shared sites were strongly enriched at genes encoding muscle structural proteins and also at a smaller set of genes encoding signalling and cell cycle proteins. In particular, Tead1 and Tead4 occupied sites at genes of the Hippo signalling pathway like *Lats2* and *Amotl2* in C2C12 cells and in mature fibres. Similarly, in post-mitotic muscle, Tead4 occupied sites at the *Ccnd1* and *Ctgf* loci that normally contribute to its proliferative function. This may reflect the known role of the Tead4-Yap1 axis in regulating muscle fibre size [13].

Despite the observed genomic occupancy, Tead4 inactivation in mature fibres had no marked effect on Pol II and H3K27ac distribution, led to only minor effects on target gene expression, and resulted in no evident phenotype. In contrast, Tead4 inactivation led to delayed muscle regeneration after notexin treatment. Significant reductions in muscle mass and fibre size were seen after 15 days, but by 30 days these parameters were comparable to those seen in control animals. In addition, Tead4 contributed to activation of muscle structural genes during regeneration-induced differentiation. The absence of phenotype in mature fibres is in agreement with a previous report where Tead4 was inactivated in post-implantation embryos [43]. Similarly, while Tead4 loss delayed regeneration, this process was not completely impaired, explaining the absence of a notable muscle phenotype seen in the study of Yagi et al., although they did not specifically assay regeneration in their Tead4 mutant animals [43]. The results obtained with differentiating PMs *in vitro* and in muscle *in vivo* are all in accordance with strong redundancy between Tead1 and Tead4 in the myogenic process that minimises the effects seen upon loss of Tead4 alone. Nevertheless, our study defines for the first time the critical roles of these factors in myogenic differentiation.

## Materials and methods

### Ethics statement

Mice were kept in accordance with the institutional guidelines regarding the care and use of laboratory animals and in accordance with National Animal Care Guidelines (European Commission directive 86/609/CEE; French decree no.87–848). All procedures were approved by the French national ethics committee.

### Mice

Intra-peritoneal injection of Tamoxifen (100μl of 1mg/ml) for four consecutive days was performed on 6–7 week-old animals. After 3 weeks, animals were sacrificed, the tibialis anterior and gastrocnemius muscles were dissected and deletion of *Tead4* was verified by PCR genotyping and RNA was prepared. For regeneration, notexin was injected in the tibialis anterior of mice previously treated with Tam. Four subsequent Tam injections were performed following notexin treatment to inactivate Tead4 in the newly forming fibres. Hind-limb grip strength was measured using a Bioseb Grip Strength Meter. Three consecutive readings were performed for each mouse within the same session and the mean value was recorded as the maximal grip strength for each mouse. Body weight was recorded using an electronic balance after sacrificing the mice. The tibialis anterior muscle was then dissected and its mass was measured. The tibialis anterior mass is represented as % of body weight. For fibre cross-section area measurements, transverse cryosections (8μm) of mouse tibialis anterior muscle were stained with hematoxylin and eosin. Slides were scanned using NanoZoomer-XR Digital slide scanner (Hamamatsu Photonics K.K.). Cross-section area was analyzed using the RoiManager plugin of Fiji image analysis software.

### Generation of *Tead4*^*lox/lox*^ mice

The conditional *Tead4* mutant allele was generated using a targeting construct where *Tead4* exons 2 and 3 were flanked by two *loxP* sites. A neomycin resistance cassette (PGK-Neo) flanked by two *Frt* sites was inserted immediately downstream of the 5’ loxP site. The targeting vector based on pKOII contained a diphtheria toxin A (DTA) counter selection cassette to enrich for homologous recombination events. Homology arms were subcloned from cosmids MPMGcPO454Q2 and MPMGc121P0454Q01 (The German Resource Center for Genome Research) using the restriction enzymes NaeI and EcoRV (3.8 kb, short arm) and EcoRV and KpnI (11.2kb, long arm). The *Tead4* targeting construct was electroporated into V6.5 F1 hybrid embryonic stem (ES) cells [44] after linearization with NotI and subjected to G418 selection. Homologous recombination events in individual ES cell clones were detected by Southern blot analysis of XbaI digested DNA using a probe located outside of the homology arms and by PCR analysis using the following primers: (5’-loxP-FW) AGTGCATGAGGCAAGAGGC, (5’-loxP-RV) GCTCCTGGGACCATAGTTA; (3’-loxP-FW) CAGGCCTCTCTCTGAGGTGA, (3’-loxP-RV) ACTATGAGAGCCTCACAGGC. A positive clone was microinjected into C57BL/6 (B6) blastocysts before transplantation into pseudopregnant foster mothers. Chimeric mice were mated to *Flp*-expressing transgenic mice to remove the neomycin resistance cassette by *Flp*-mediated recombination leaving behind a single Flp site and two loxP sites flanking exon 2 and 3. These mice were the bred with previously described *Hsa*::CreER^T2^ mice [32].

### Cell culture, differentiation and transfections

C2C12 cells were grown in 20% foetal calf serum (FCS) containing DMEM medium and were differentiated for most experiments up to six days in 2% horse serum (HS) containing DMEM medium. Adult mouse primary myoblasts were isolated from C57BL/6 wild type 3–4 week-old mice and plated on matrigel-coated dishes. The primary myoblasts were grown in IMDM GLUTAMAX-I medium with 20% FCS and were differentiated in the same medium with 2% HS. The siRNA transfection experiments were performed as per the Lipofectamine RNAiMAX manufacturer’s protocol and cells were harvested at indicated time points of differentiation after the siRNA transfection. ON-TARGET-plus SMARTpool siRNAs for Tead1, Tead2 and Tead4 knockdown and non-targeting siRNA were purchased from Dharmacon Inc. (Chicago, Il., USA). The siRNA experiments were performed at least in triplicates. Phase contrast images were taken at 4x magnification using the EVOS digital microscope.

### Antibodies and primers

A list of all antibodies and primers used can be found in S6 Dataset.

### Immunoblotting

Whole cell extracts were prepared by the standard freeze-thaw technique using LSDB 500 buffer (500 mM KCl, 25 mM Tris at pH 7.9, 10% glycerol, 0.05% NP-40, 1 mM DTT, and protease inhibitor cocktail) and Immunoblotting was performed by standard procedure.

### Immunofluorescence and fusion index

1x10^5^ cells were seeded on coverslips in 35mm dishes with matrigel for primary myoblasts and without matrigel for C2C12 cells and were transfected with siRNA 4 hours after seeding. Cells were refreshed 6 to 8 hours after the siRNA treatment and fixed on day 6 of differentiation with 4% formaldehyde for 10 mins. Cells were washed with PBS and permeabilized with 0.5% triton for 10 mins, washed twice with PBS-tween 0.2% and blocked with 5% BSA for 30 minutes. Cells were incubated with primary antibody overnight at 4°C followed by three PBS-tween 0.2% washes. Secondary antibody incubation was done for 30 minutes at room temperature. Cells were washed thrice with PBS-tween 0,2% and stained with DAPI. Coverslips were mounted on superfrost glass slides using Vectashield. Slides were visualised using an inverted fluorescence microscope at 10x magnification in all experiments.

To quantify the fusion in double and triple knockdown experiments, we calculated the fusion index as the percentage of number of nuclei within the Myh-positive cells above total number of nuclei counted in a field. Nuclei in fields from three replicate experiments were counted and analysed by a two-tailed t-test. Note that Myh positive cells with only 3 nuclei were taken for the counting of the nuclei.

### RNA extraction, RT-qPCR and RNA-sequencing

Total RNA was extracted using the GenElute Mammalian Total RNA Miniprep Kit from Sigma. cDNA was prepared with using SuperScript II Reverse Transcriptase (RT) using the kit protocol and quantitative PCR was carried out with the SYBR Green I (Qiagen) and monitored using the Roche Lightcycler 480. Primer sequences were designed using Primer3plus software and beta-actin was used as normalization control.

Messenger-RNA-seq was performed essentially as described [45, 46]. Sequence reads mapped to reference genome mm9/NCBI37 using Tophat [47]. Data normalization and quantification of gene expression was performed using the DESeq 2 Bioconductor package [48]. Significantly deregulated genes were selected using a log2 fold change >1 and <1 and adjusted p-value <0,05. Gene ontology analyses were performed using the DAVID functional annotation clustering tool available at the website- https://david.ncifcrf.gov/. For GSEA analyses, we used the mean of the log2 fold changes of the biological replicates as metric for the H Hallmark gene sets of the BROAD javaGSEA tool with 1,000 permutations and the canonical pathway (cp) subcollection of the C2 curated BROAD molecular signature gene-set collection.

### ChIP and ChIP-sequencing

Chromatin immunoprecipitation from C2C12 cells was performed by standard procedures as previously described [45] [49]. For ChIP from mouse muscle, muscles harvested from hind limbs of three adult *Hsa*::Cre-ER^T2^::*Tead4*^lox/lox^ mice with or without Tamoxifen injection were either snap frozen or immediately used for ChIP. The tissue was minced and quickly homogenised in cold hypotonic buffer with protease inhibitors using an Ultraturax homogeniser. The homogenised tissue lysate was fixed with 1% formaldehyde in fresh hypotonic buffer for 10 mins shaking at room temperature. Fixation was stopped by adding glycine to 0.15M concentration. Lysate was centrifuged at 3000rpm 5 mins at 4°C and the pellet was resuspended in fresh hypotonic buffer (25 mM HEPES, pH 7.8, 1.5 mM MgCl2, 10 mM KCl, and 0.1% NP-40), supplemented with Protease Inhibitor Cocktail (Roche, Basel, Switzerland). Lysate was filtered to eliminate debris and nuclei were collected using cell strainer of 70 μm pore size. The filtrate was centrifuged for 5 mins at 3000rpm to obtain a nuclear pellet that was resuspended and incubated 10 min at 4°C in sonication buffer (EDTA 10mM, Tris-HCl, pH 8.0, 50mM, SDS 1% with protease inhibitor cocktail and PMSF) and then sonicated using Covaris sonicator for 20 to 25 mins. Lysate was then centrifuged for 15 mins at 11000g at 4°C to obtain the chromatin supernatant fraction that was the used for ChIP. ChIP-seq libraries were prepared and sequenced on an Illumina Hi-seq2500 as single-end 50-base reads. After sequencing, peak detection was performed using the MACS software [50] http://liulab.dfci.harvard.edu/MACS/). Global clustering, meta-analyses and quantitative comparisons were performed using seqMINER and R (http://www.r-project.org/). Peaks were annotated with Homer (http://homer.salk.edu/homer/ngs/annotation.html) using a window of ±50 kb (or as nearest gene) relative to the transcription start site of RefSeq transcripts. *De novo* motif discovery was performed on the 200 base pairs surrounding the top 600 Tead1 and Tead4 peaks using MEME-ChIP. Motif enrichment analyses were performed using in house algorithms as described [49].

### Accession numbers

The public data for H3K27ac and H3K4me3 data were taken from the GEO accession GSE25308 [51], Jun GSE37525, Srf, GSM915168, Runx, GSM1354734. Myod1 and Myog ChIP-seq raw data were from GSE44824 [27] and re-analyzed in parallel to the Tead4 and Tead1 ChIP-seq data. The data in this paper have been assigned the accession number GSE82193 in the GEO database.

## Supporting information

S1 Fig Tead1 and Tead4 expression in PMs and C2C12 cells. **A-B.** Immunoblots showing Tead1 or Tead4 protein levels in differentiating PMs (A) and C2C12 cells (B) transfected with the indicated siRNAs. Beta-actin is used as loading control. **C** Immunostaining for Tead1 and Tead4 in non-differentiated (ND) and differentiated C2C12 cells. Differentiated cells were counterstained with Myh to identify myotubes. Arrows indicate the nuclei of differentiated myotubes. **D.** Immunostaining for Tead1 and Tead4 in differentiated PMs. In the centre and right panels, cells were counterstained with Dmd to identify myotubes. The left panel shows a region where differentiated myotubes and non-differentiated PMs were intermixed allowing a comparison of the localisation of the proteins in the two states. All scale bars 100 μm.
(TIF)

S2 Fig Tead4 occupancy in C2C12 cells. **A**. UCSC genome browser shot of Tead4 occupancy at the *Ctgf*, *Ccnd1* and *Acta1* loci in differentiated and non-differentiated C2C12 cells. The Tead4-bound sites are indicated with arrows. **B**. Read density maps showing comparison of Tead4 binding with that of Jun, Runx and Srf.
(TIF)

S3 Fig Tead1 genomic occupancy in C2C12 cells. **A.** Localisation of Tead1 occupied sites in non-differentiated C2C12 cells relative to genomic annotations and the TSS. **B.** Results of MEME analysis on the top 600 Tead1 occupied sites in non-differentiated C2C12 cells. Lower panel indicates the frequency of occurrence of DNA binding motifs for the indicated transcription factors at Tead1 occupied sites comparing the expected and observed values. **C.** Localisation of Tead1 occupied sites in differentiated C2C12 cells. **D.** UCSC genome browser view of Tead1 occupancy at the *Ankrd1* and *Vdr* loci in the non-differentiated and differentiated state. **E**. Read density cluster map to compare Tead1 and Tead4 occupancy in non-differentiated cells.
(TIF)

S4 Fig Transcription factor occupancy at the *Tead1* and *Tead4* gene loci. **A-B.** UCSC screenshots showing Tead4 and Tead1 occupancy and H3K27ac at *Tead1* and *Tead4* gene loci in non-differentiated and differentiated C2C12 cells along with Myog and Myod1 occupancy in differentiated cells.
(TIF)

S5 Fig Myog regulates Tead4 and Mef2c expression. **A.** Immunostaining for Myh expression to show inhibition of C2C12 and PM differentiation following siMyog. **B.** RT-qPCR analyses of gene expression in siControl and siMyog C2C12 cells. **C.** UCSC screenshots showing Tead4 and Myog occupancy and H3K27ac at the *Mef2c* locus in differentiated C2C12 cells. Arrows indicate Tead4 or Myog bound sites that co-localise and/or co-localise with H3K27ac in differentiated cells.
(TIF)

S6 Fig Integration of Tead1 genomic occupancy with chromatin modifications. **A.** Read density cluster map showing chromatin modifications at Tead1-occupied sites in non-differentiated cells. **B.** Venn diagrams illustrating the overlap of chromatin modifications with Tead1 genomic occupancy. **C.** Identification and ontology analysis of genes associated with Tead4 sites at active H3K27ac marked regulatory elements. **D.** UCSC screenshots showing Tead1, Tead4 occupancy and H3K4me3 and H3K27ac at a selection of loci illustrating constitutive and acquired chromatin marks and Tead binding during differentiation.
(TIF)

S7 Fig Sites co-occupied by Tead4, Myod1 and Myog. **A.** Read density cluster maps showing sites occupied by Myog, Myod1 and Tead4 in differentiated C2C12 cells. The metaprofiles of selected clusters are shown to the right. **B.** Read density cluster map comparing sites occupied by Myog and Myod1 in differentiated cells with Tead1 in non-differentiated cells. Only a small set of common sites was identified. **C.** Frequency of occurrence of transcription factor binding motifs at the commonly occupied sites from panel A. **D.** Venn diagrams illustrating the overlap of genes associated with Tead4, Myod1 and Myog bound sites. **E-F.** Read density cluster maps showing sites co-occupied by Tead4 or Tead1 and Mef2a. The metaprofiles of selected clusters are shown to the right.
(TIF)

S8 Fig Gene expression programs in C2C12 cells and PMs. **A-B.** Venn diagrams illustrating the overlap of up and down-regulated genes in differentiating PMs and C2C12 cells. The ontology analyses of the commonly regulated genes of both categories are shown.
(TIF)

S9 Fig Gene expression in differentiating C2C12 cells. **A.** Classification of gene expression changes into classes with different kinetics. **B.** GSEA analyses of genes up and down-regulated during C2C12 cell differentiation. The most significant categories are shown.
(TIF)

S10 Fig Gene expression in differentiating PMs. **A.** Classification of gene expression changes into classes with different kinetics. **B.** GSEA analyses of genes up and down-regulated during PM differentiation. The most significant categories are shown.
(TIF)

S11 Fig Genes regulated by siTead1/4 silencing in PMs and C2C12 cells. **A.** Venn diagram representing genes specifically or commonly down-regulated in C2C12 cells and PMs along with their BP-FAT ontology. **B.** Venn diagram representing genes specifically or commonly up-regulated in C2C12 cells and PMs along with their BP-FAT ontology.
(TIF)

S12 Fig Tead genome occupancy in muscle. **A.** Read density maps comparing Tead4 occupancy in muscle with that of Pol II and H3K27ac. The ontology of the genes associated with the subset of co-localising sites is indicated. **B.** Read density maps comparing Tead1 and Tead4 occupancy in muscle. The ontology of the genes associated with the subset of co-localising sites is indicated.
(TIF)

S13 Fig Tead occupancy in muscle in vivo. UCSC genome browser view of the *Acta1* locus showing Tead1, Tead4, Pol II and H3K27ac ChIP-seq from WT and MT muscle. The arrow indicates the major Tead1/4 binding site.
(TIF)

S14 Fig Tead1/4 genome occupancy in C2C12 cells *in vitro* and in muscle *in vivo*. UCSC screenshots of the *Amotl2* locus showing Tead1, Tead4, Myod and Myog occupancy and H3K27ac in differentiated C2C12 cells along with Tead1, Tead4, H3K27ac and Pol II in WT and MT muscle. Arrows indicate Tead1/4 bound sites common to both C2C12 cells and muscle.
(TIF)

S15 Fig Tead4 inactivation in mature muscle fibre. **A**. Outline of experimental protocol showing days of Tam injection. **B**. RT-qPCR of the indicated genes. T-test; p-value * <0,05. N = 3. **C.** Tibialis anterior cross-section area of the indicated animals. Two-way anova, no significant values. N = 4. **D.** Tibialis anterior mass of the indicated animals. Two-tailed t-test, no-significant values. **E**. Grip strength of the indicated animals. no-significant values.
(TIF)

S1 Dataset Coordinates of Tead genome occupancy. MACS ouput files with peak coordinates of Tead1 and Tead4 Chip-seq.
(XLSX)

S2 Dataset Tead4 and Myog/MyoD1 colocalisation. Peak coordinates of Tead4 sites co-localising with Myog and Myod1.
(XLSX)

S3 Dataset Tead regulated genes in C2C12 cells. Genes de-regulated by siTead1/4 silencing in C2C12 cells.
(XLSX)

S4 Dataset Tead regulated genes in PMs. Genes de-regulated by siTead1/4 silencing in PMs.
(XLSX)

S5 Dataset Ontology of Tead-regulated genes. Ontology analyses of genes commonly or specifically regulated by si*Tead1/4* silencing in PMs and C2C12 cells.
(XLSX)

S6 Dataset Antibodies and primers used in this study. (XLSX)
