# Supplementary figures and images for: TEAD transcription factors are required for normal primary myoblast differentiation in vitro and muscle regeneration in vivo
Source: PLoS Genet. 2017 Feb 8;13(2):e1006600. doi: 10.1371/journal.pgen.1006600 (PMC5323021; doi:10.1371/journal.pgen.1006600)

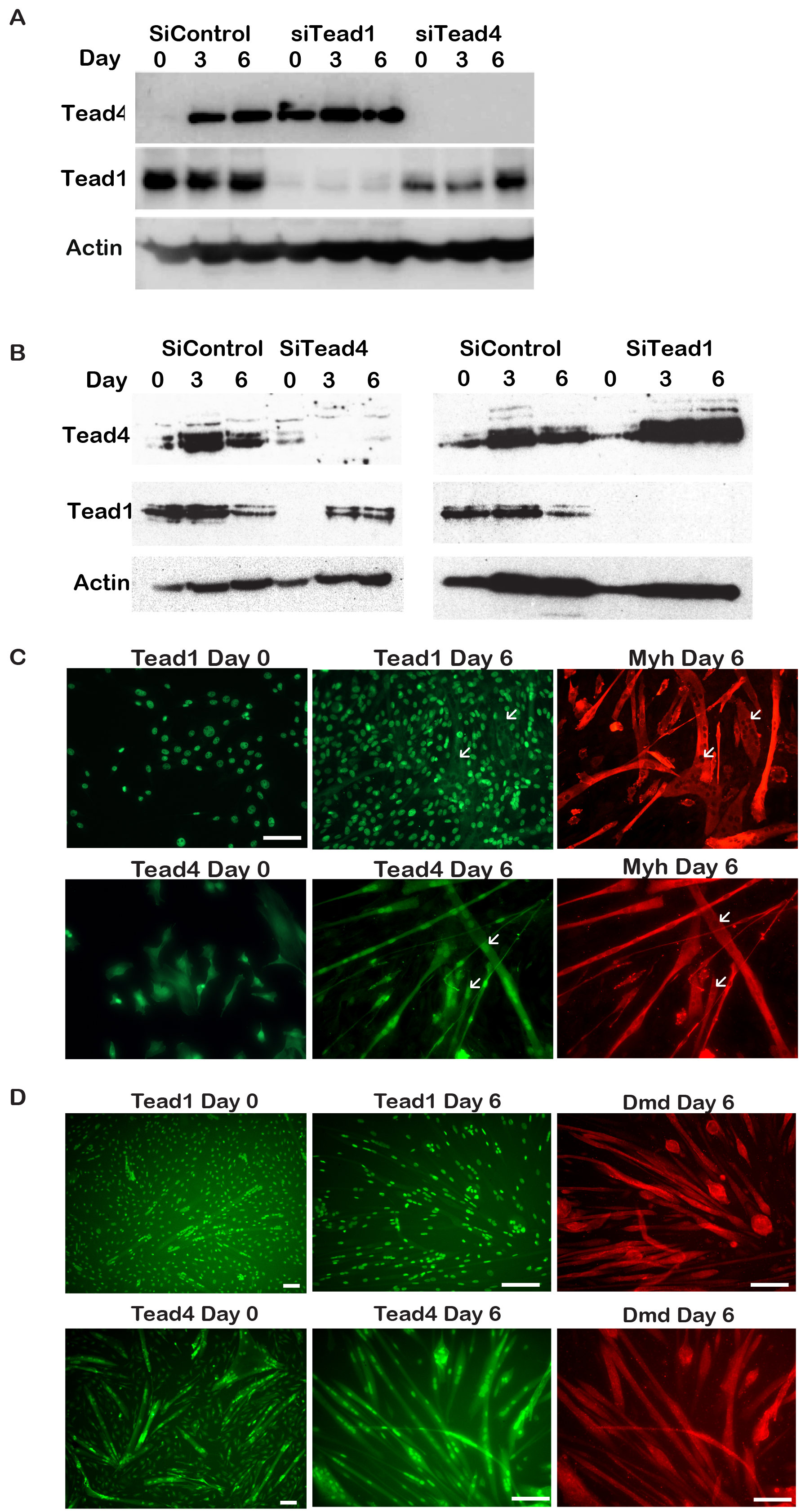

Supplement: S1 Fig — A-B. Immunoblots showing Tead1 or Tead4 protein levels in differentiating PMs (A) and C2C12 cells (B) transfected with the indicated siRNAs. Beta-actin is used as loading control. C Immunostaining for Tead1 and Tead4 in non-differentiated (ND) and differentiated C2C12 cells. Differentiated cells were counterstained with Myh to identify myotubes. Arrows indicate the nuclei of differentiated myotubes. D. Immunostaining for Tead1 and Tead4 in differentiated PMs. In the centre and right panels, cells were counterstained with Dmd to identify myotubes. The left panel shows a region where differentiated myotubes and non-differentiated PMs were intermixed allowing a comparison of the localisation of the proteins in the two states. All scale bars 100 μm. (TIF) [file pgen.1006600.s001.tif]

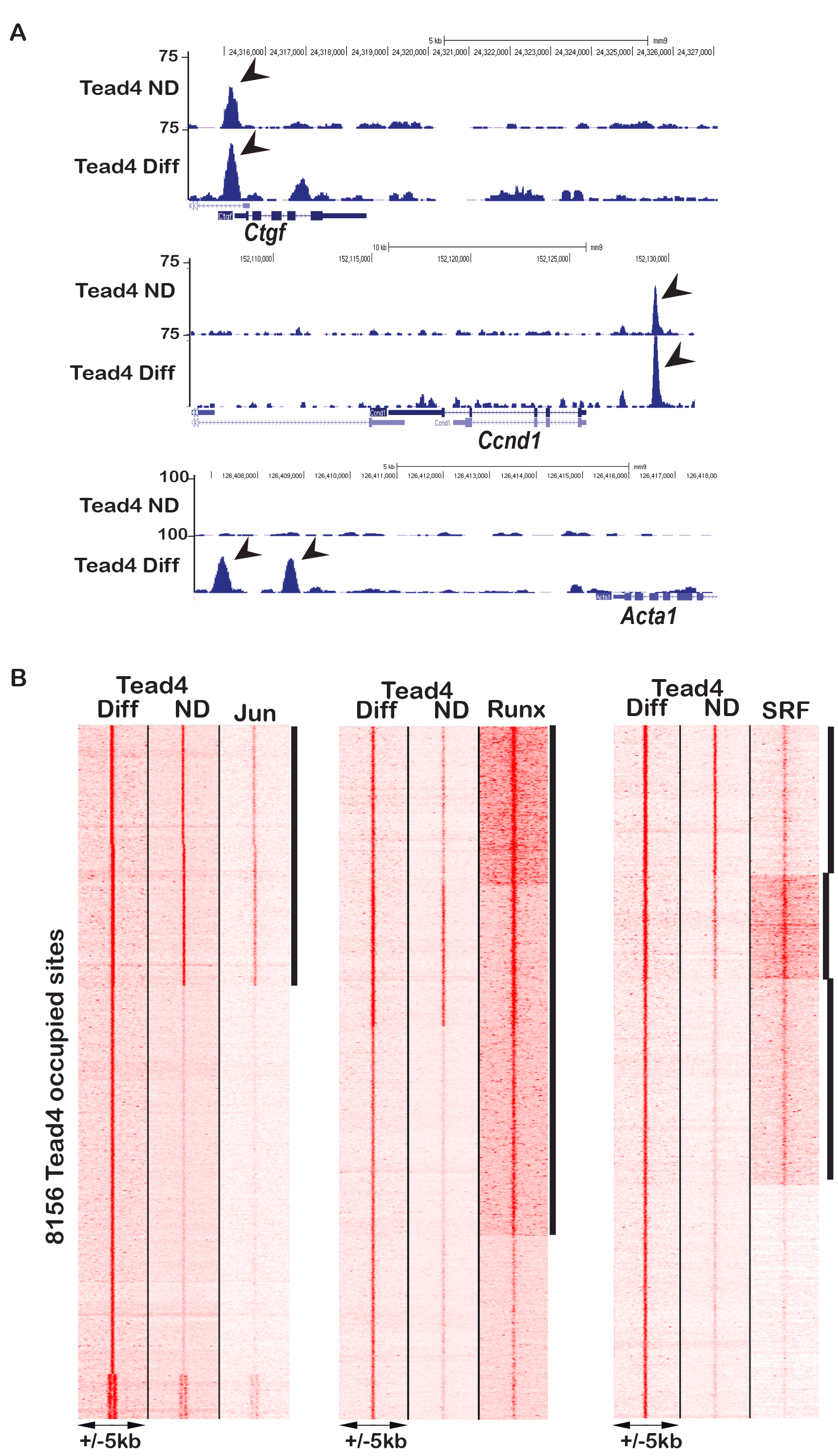

Supplement: S2 Fig — A. UCSC genome browser shot of Tead4 occupancy at the Ctgf, Ccnd1 and Acta1 loci in differentiated and non-differentiated C2C12 cells. The Tead4-bound sites are indicated with arrows. B. Read density maps showing comparison of Tead4 binding with that of Jun, Runx and Srf. (TIF) [file pgen.1006600.s002.tif]

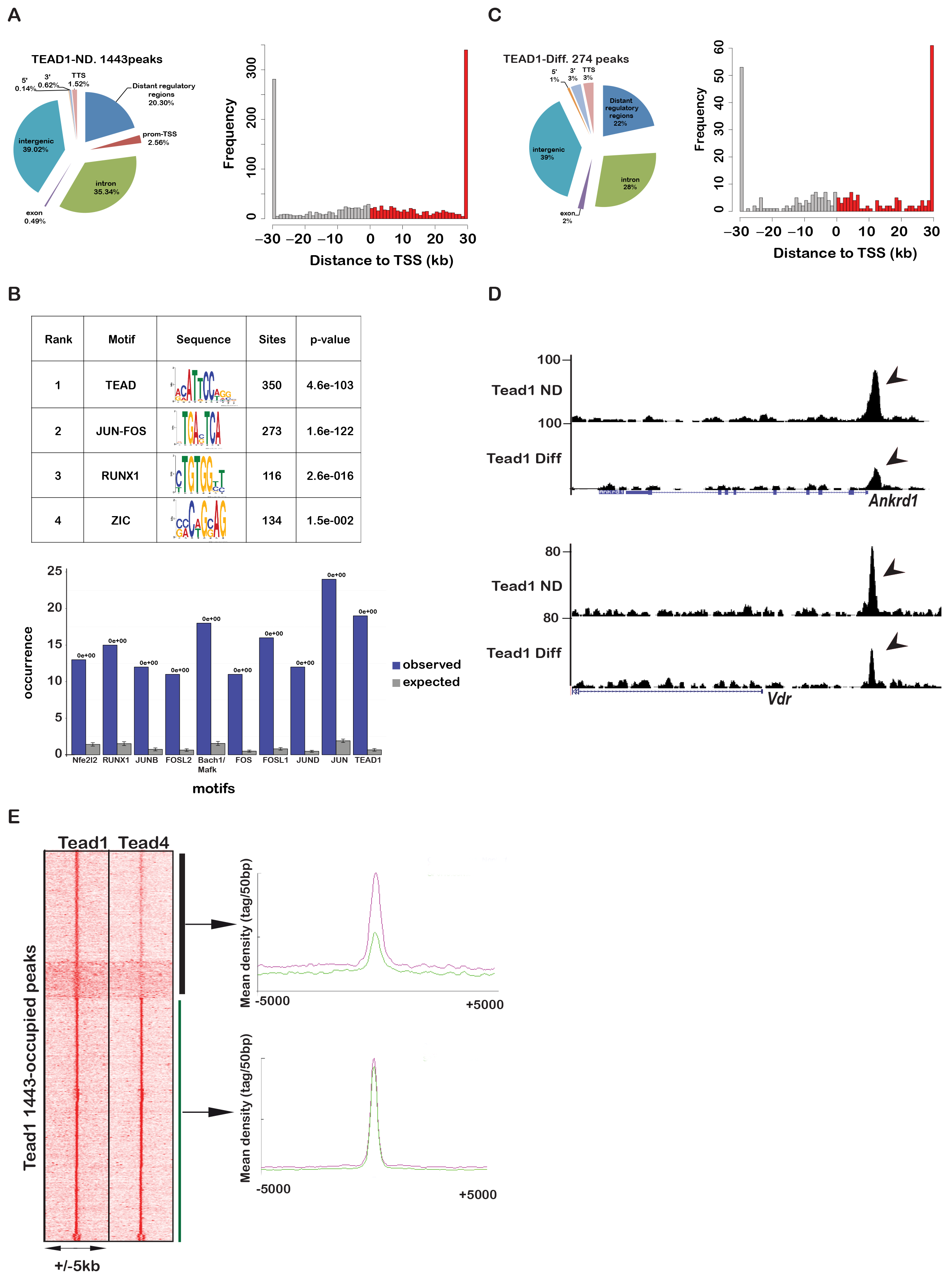

Supplement: S3 Fig — A. Localisation of Tead1 occupied sites in non-differentiated C2C12 cells relative to genomic annotations and the TSS. B. Results of MEME analysis on the top 600 Tead1 occupied sites in non-differentiated C2C12 cells. Lower panel indicates the frequency of occurrence of DNA binding motifs for the indicated transcription factors at Tead1 occupied sites comparing the expected and observed values. C. Localisation of Tead1 occupied sites in differentiated C2C12 cells. D. UCSC genome browser view of Tead1 occupancy at the Ankrd1 and Vdr loci in the non-differentiated and differentiated state. E. Read density cluster map to compare Tead1 and Tead4 occupancy in non-differentiated cells. (TIF) [file pgen.1006600.s003.tif]

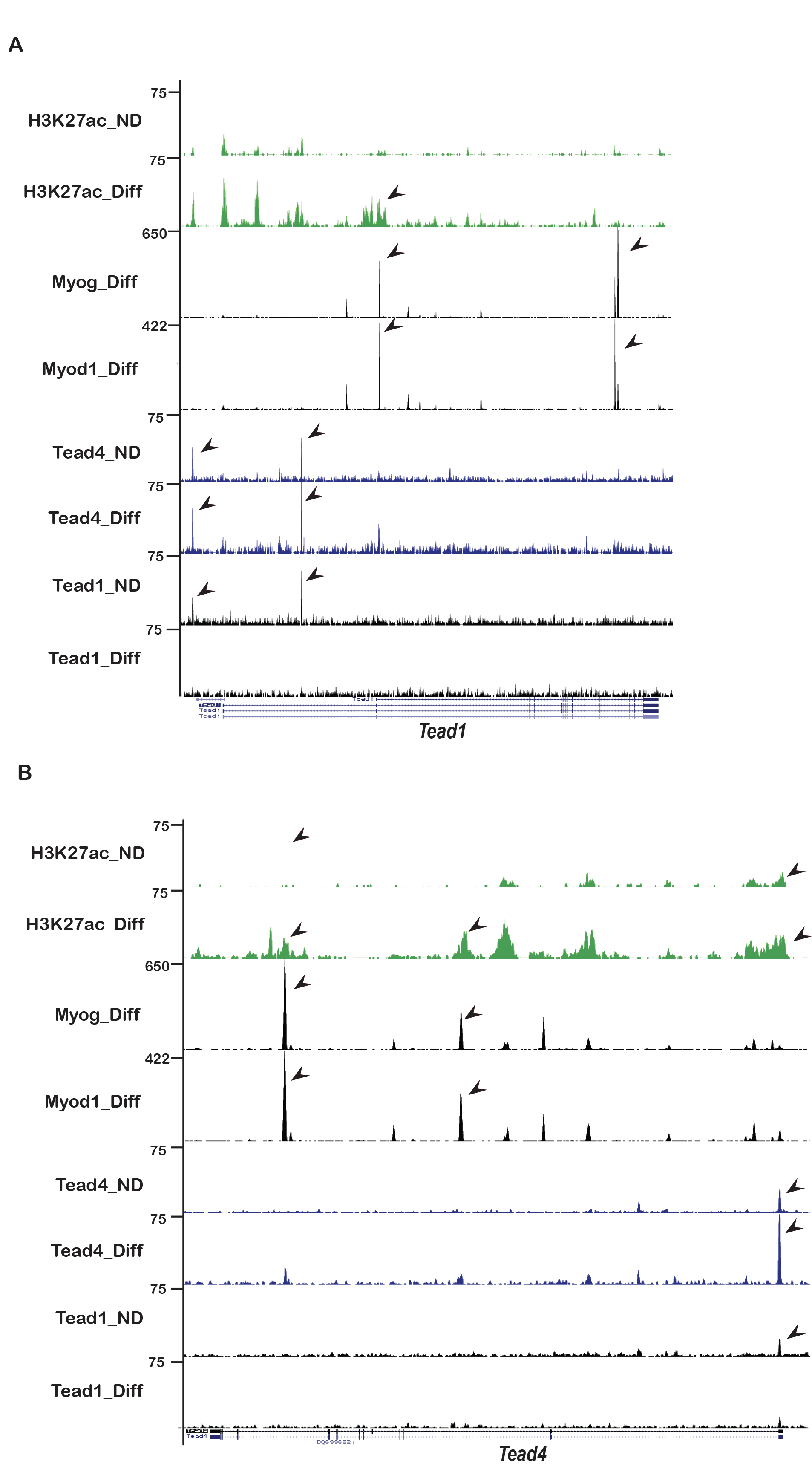

Supplement: S4 Fig — A-B. UCSC screenshots showing Tead4 and Tead1 occupancy and H3K27ac at Tead1 and Tead4 gene loci in non-differentiated and differentiated C2C12 cells along with Myog and Myod1 occupancy in differentiated cells. (TIF) [file pgen.1006600.s004.tif]

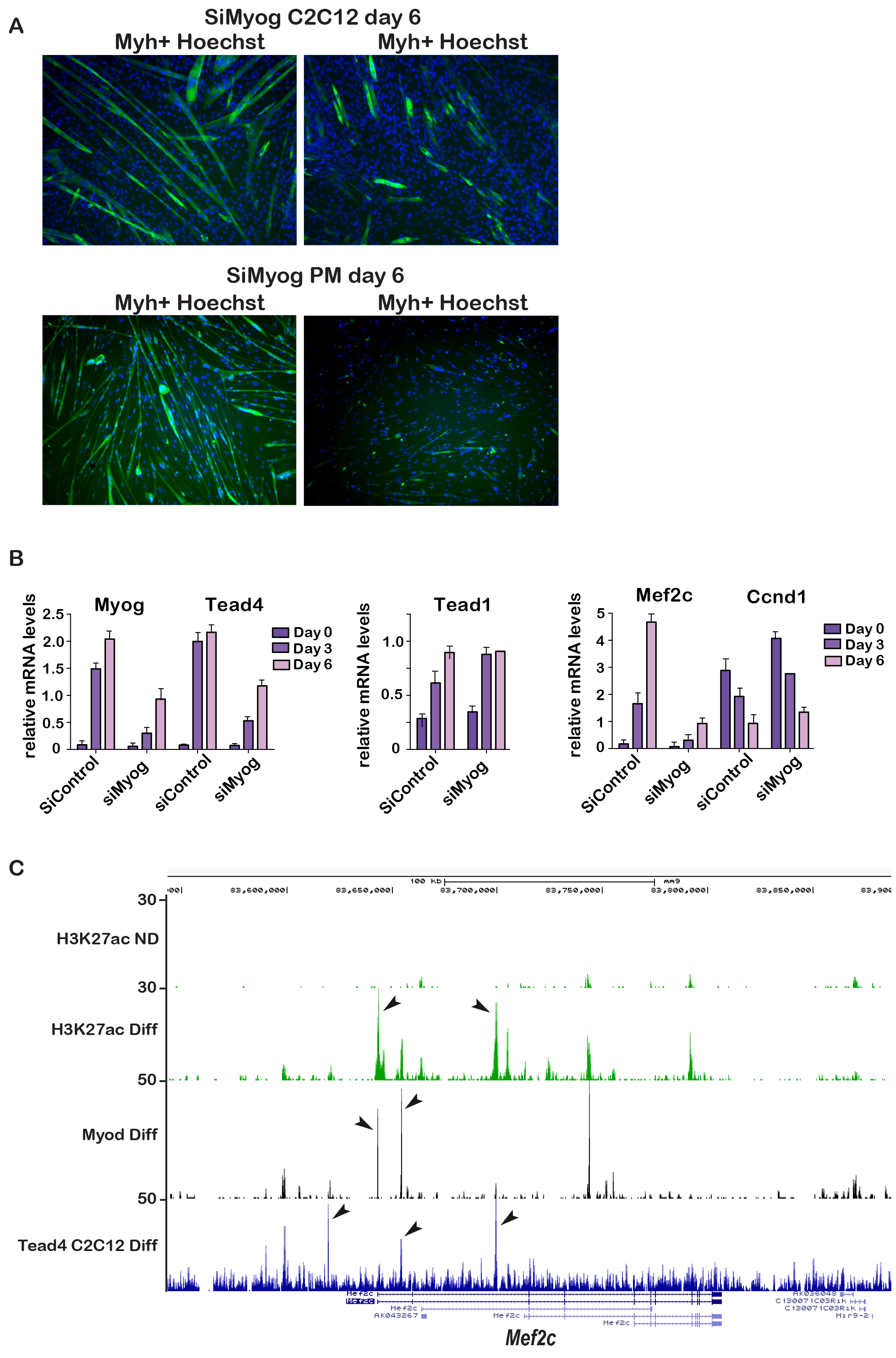

Supplement: S5 Fig — A. Immunostaining for Myh expression to show inhibition of C2C12 and PM differentiation following siMyog. B. RT-qPCR analyses of gene expression in siControl and siMyog C2C12 cells. C. UCSC screenshots showing Tead4 and Myog occupancy and H3K27ac at the Mef2c locus in differentiated C2C12 cells. Arrows indicate Tead4 or Myog bound sites that co-localise and/or co-localise with H3K27ac in differentiated cells. (TIF) [file pgen.1006600.s005.tif]

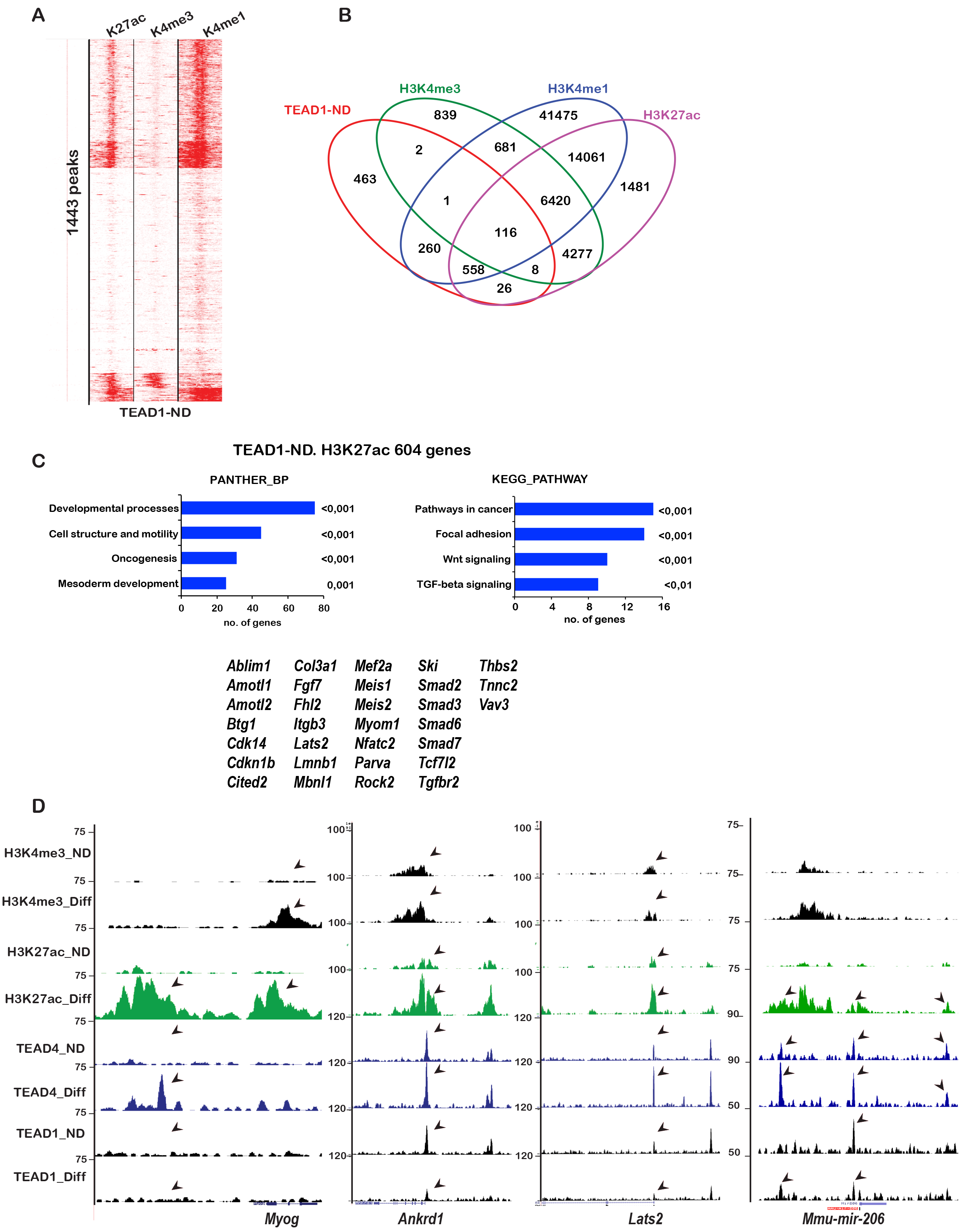

Supplement: S6 Fig — A. Read density cluster map showing chromatin modifications at Tead1-occupied sites in non-differentiated cells. B. Venn diagrams illustrating the overlap of chromatin modifications with Tead1 genomic occupancy. C. Identification and ontology analysis of genes associated with Tead4 sites at active H3K27ac marked regulatory elements. D. UCSC screenshots showing Tead1, Tead4 occupancy and H3K4me3 and H3K27ac at a selection of loci illustrating constitutive and acquired chromatin marks and Tead binding during differentiation. (TIF) [file pgen.1006600.s006.tif]

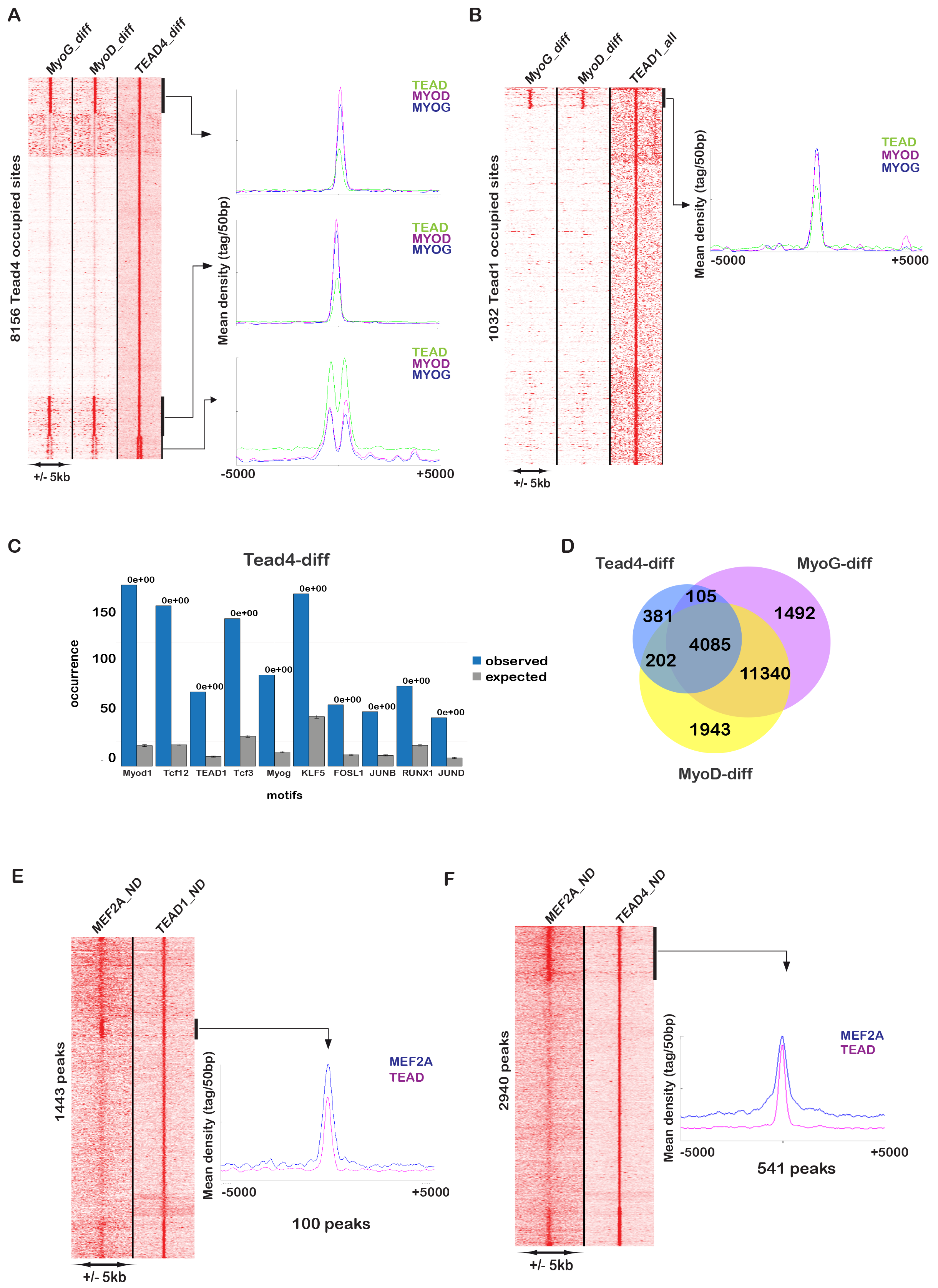

Supplement: S7 Fig — A. Read density cluster maps showing sites occupied by Myog, Myod1 and Tead4 in differentiated C2C12 cells. The metaprofiles of selected clusters are shown to the right. B. Read density cluster map comparing sites occupied by Myog and Myod1 in differentiated cells with Tead1 in non-differentiated cells. Only a small set of common sites was identified. C. Frequency of occurrence of transcription factor binding motifs at the commonly occupied sites from panel A. D. Venn diagrams illustrating the overlap of genes associated with Tead4, Myod1 and Myog bound sites. E-F. Read density cluster maps showing sites co-occupied by Tead4 or Tead1 and Mef2a. The metaprofiles of selected clusters are shown to the right. (TIF) [file pgen.1006600.s007.tif]

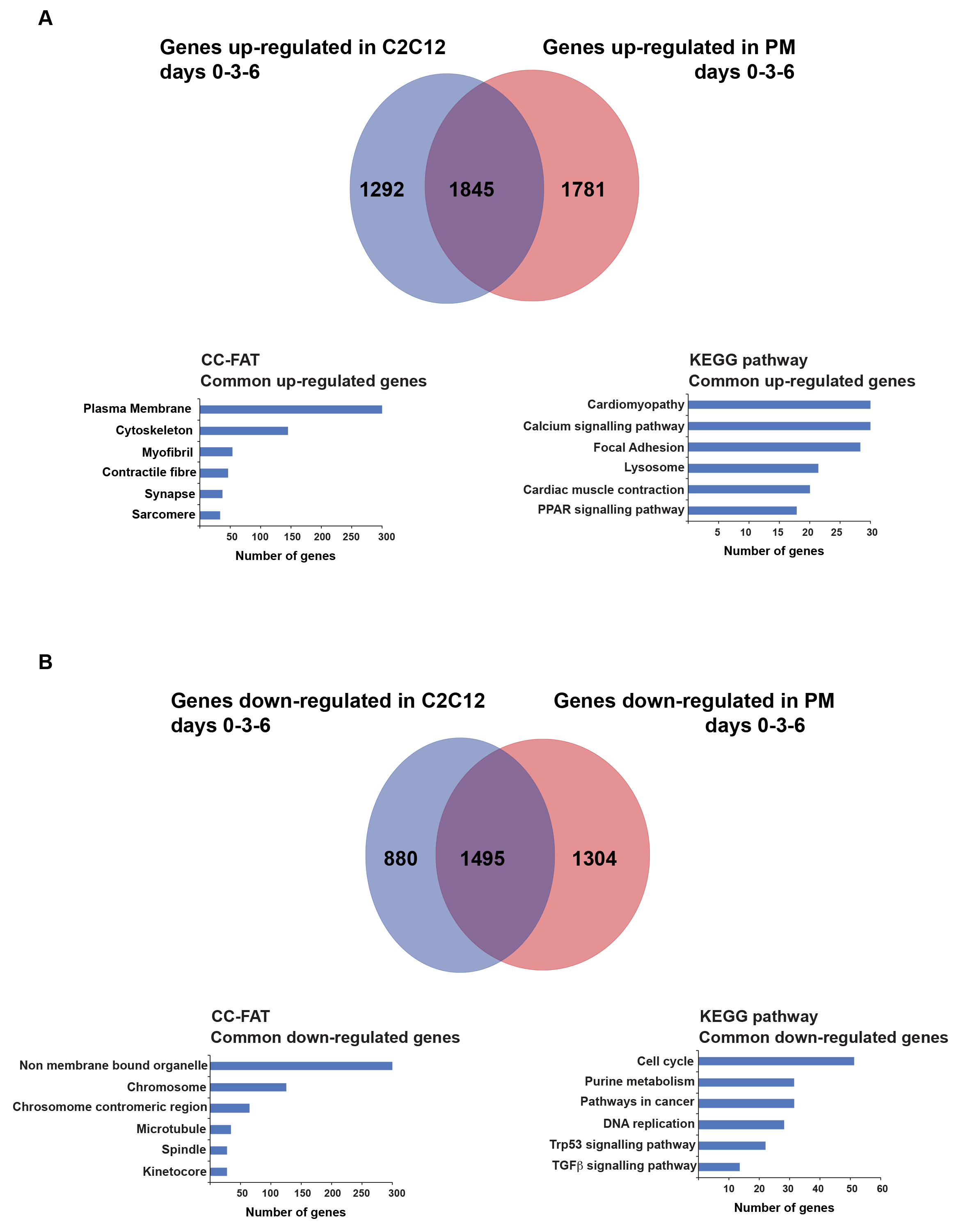

Supplement: S8 Fig — A-B. Venn diagrams illustrating the overlap of up and down-regulated genes in differentiating PMs and C2C12 cells. The ontology analyses of the commonly regulated genes of both categories are shown. (TIF) [file pgen.1006600.s008.tif]

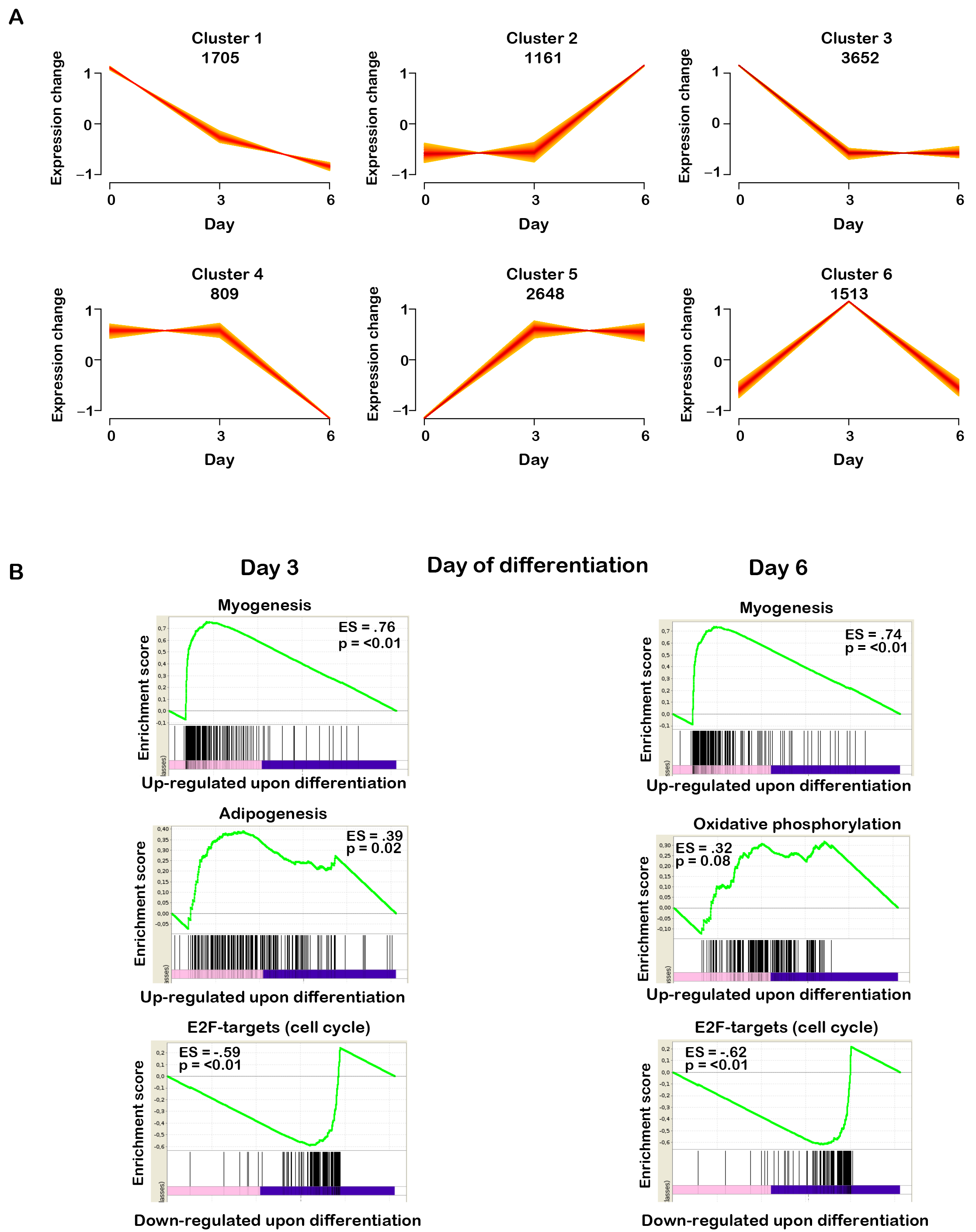

Supplement: S9 Fig — A. Classification of gene expression changes into classes with different kinetics. B. GSEA analyses of genes up and down-regulated during C2C12 cell differentiation. The most significant categories are shown. (TIF) [file pgen.1006600.s009.tif]

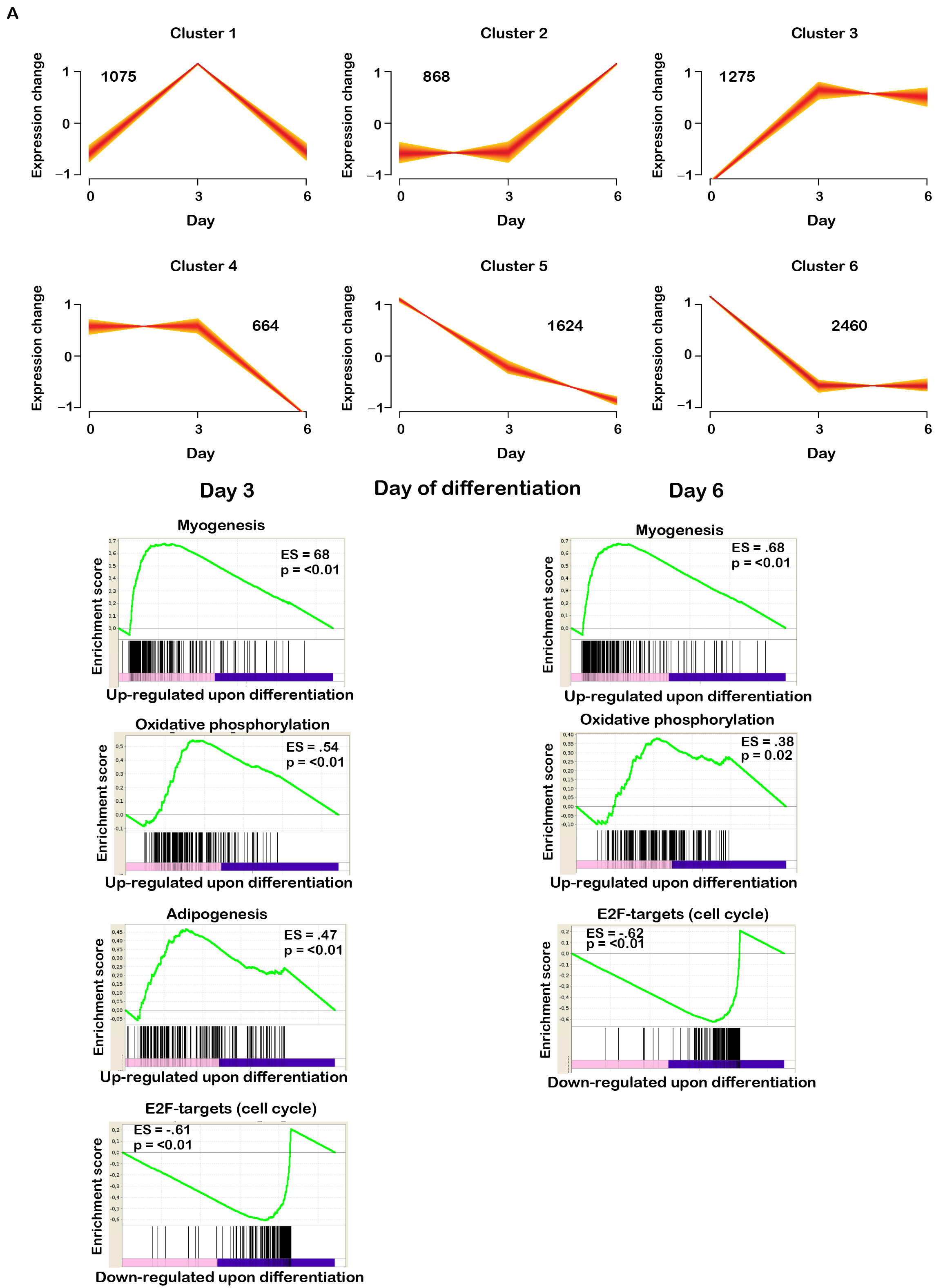

Supplement: S10 Fig — A. Classification of gene expression changes into classes with different kinetics. B. GSEA analyses of genes up and down-regulated during PM differentiation. The most significant categories are shown. (TIF) [file pgen.1006600.s010.tif]

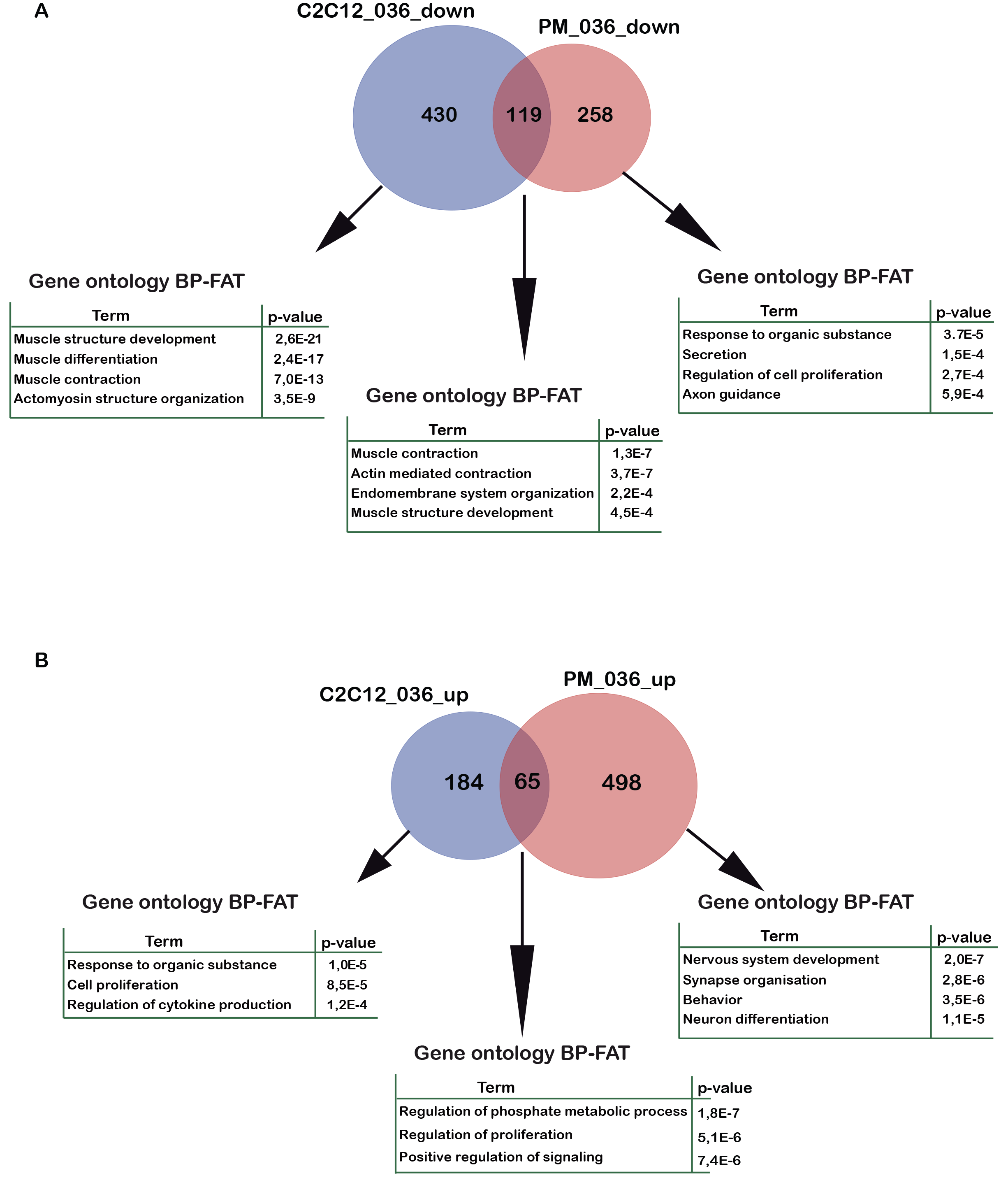

Supplement: S11 Fig — A. Venn diagram representing genes specifically or commonly down-regulated in C2C12 cells and PMs along with their BP-FAT ontology. B. Venn diagram representing genes specifically or commonly up-regulated in C2C12 cells and PMs along with their BP-FAT ontology. (TIF) [file pgen.1006600.s011.tif]

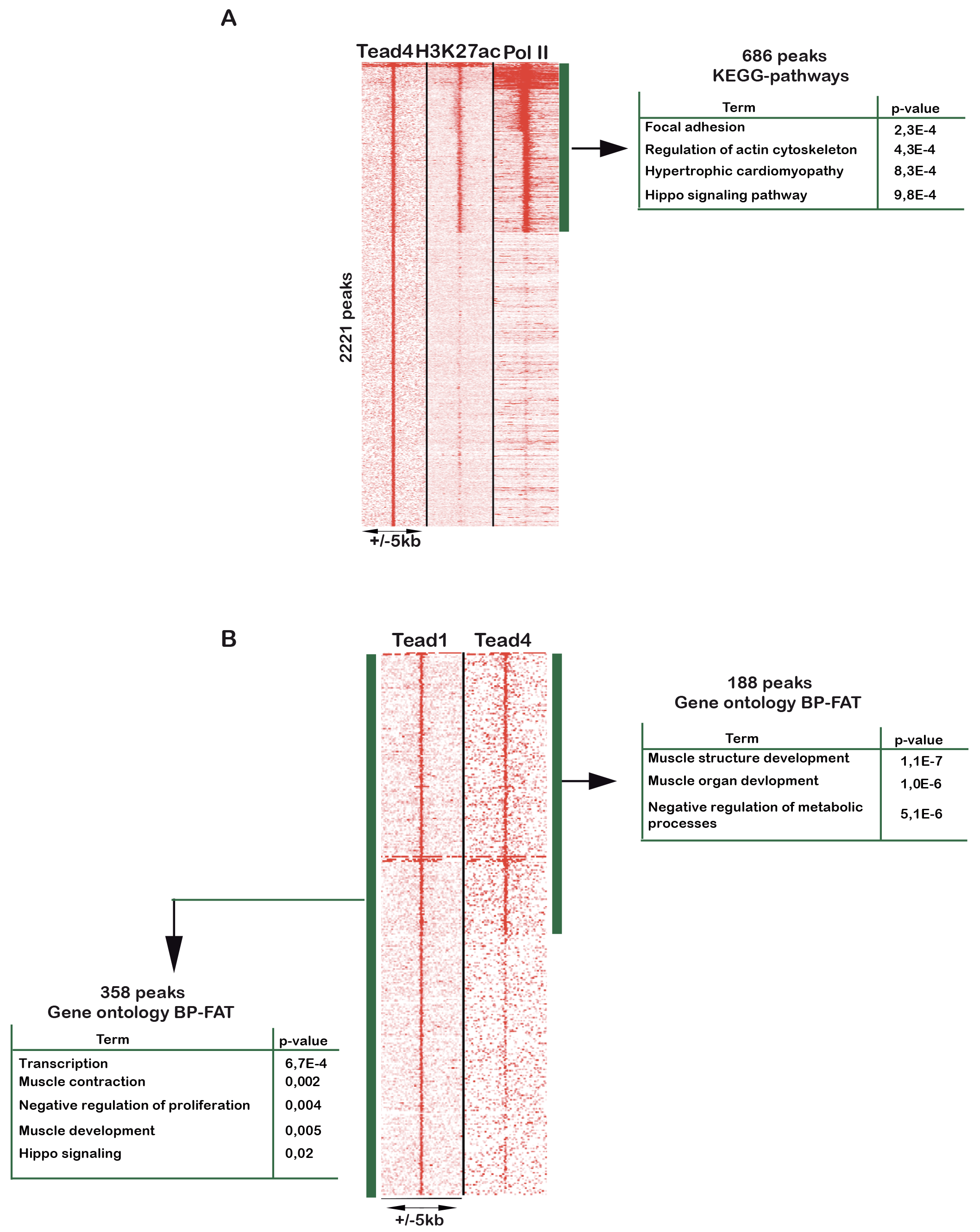

Supplement: S12 Fig — A. Read density maps comparing Tead4 occupancy in muscle with that of Pol II and H3K27ac. The ontology of the genes associated with the subset of co-localising sites is indicated. B. Read density maps comparing Tead1 and Tead4 occupancy in muscle. The ontology of the genes associated with the subset of co-localising sites is indicated. (TIF) [file pgen.1006600.s012.tif]

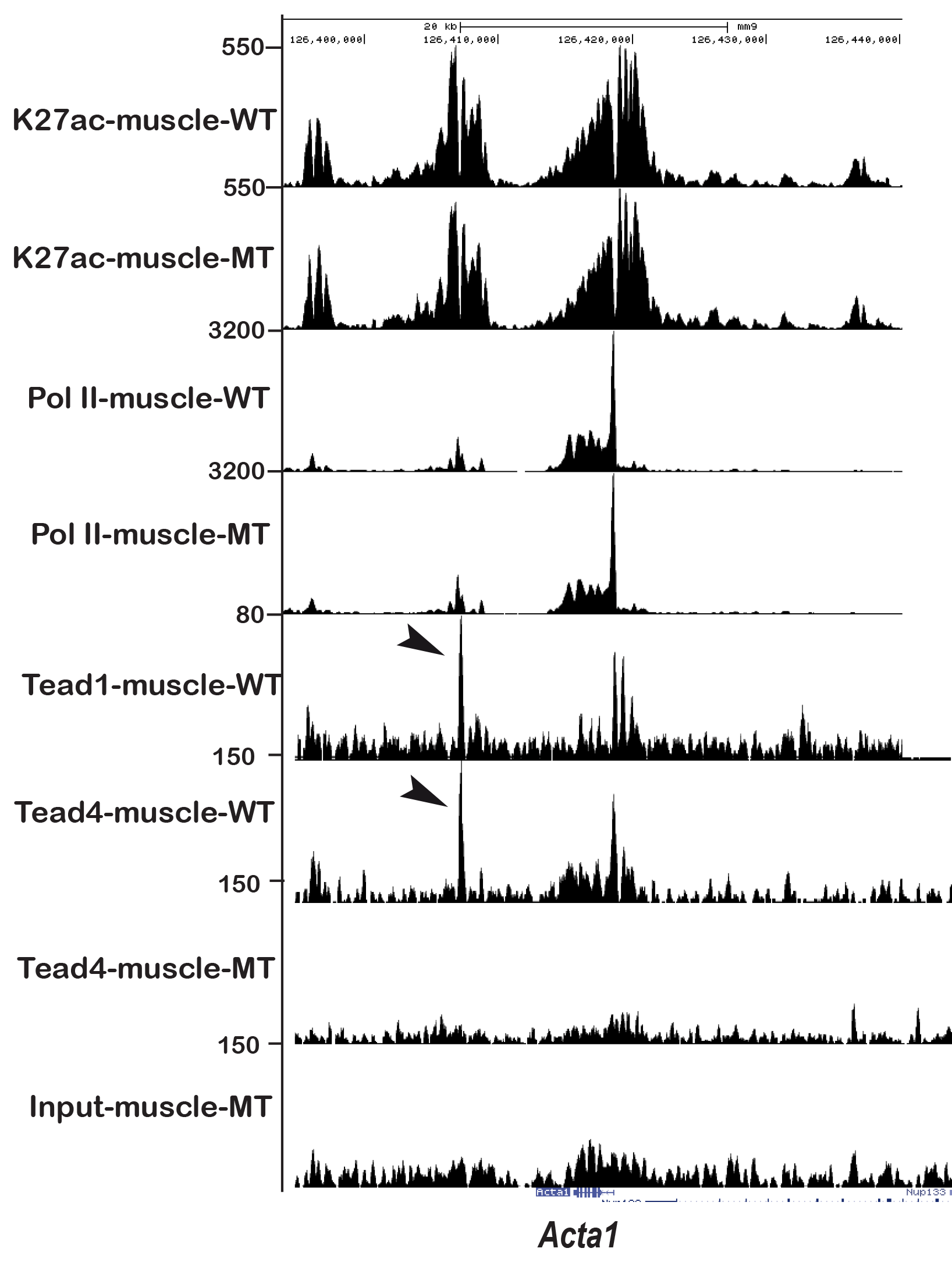

Supplement: S13 Fig — UCSC genome browser view of the Acta1 locus showing Tead1, Tead4, Pol II and H3K27ac ChIP-seq from WT and MT muscle. The arrow indicates the major Tead1/4 binding site. (TIF) [file pgen.1006600.s013.tif]

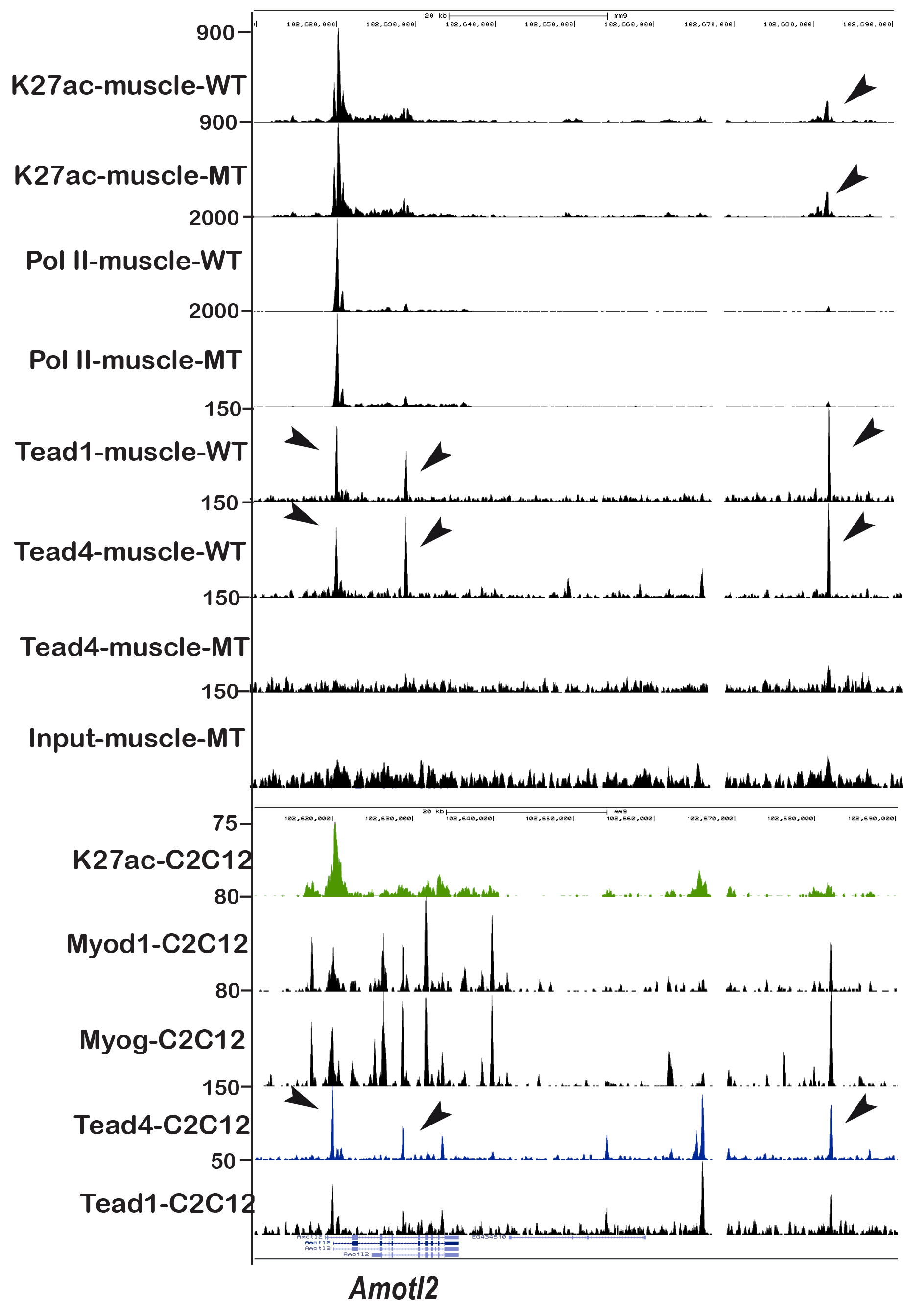

Supplement: S14 Fig — UCSC screenshots of the Amotl2 locus showing Tead1, Tead4, Myod and Myog occupancy and H3K27ac in differentiated C2C12 cells along with Tead1, Tead4, H3K27ac and Pol II in WT and MT muscle. Arrows indicate Tead1/4 bound sites common to both C2C12 cells and muscle. (TIF) [file pgen.1006600.s014.tif]

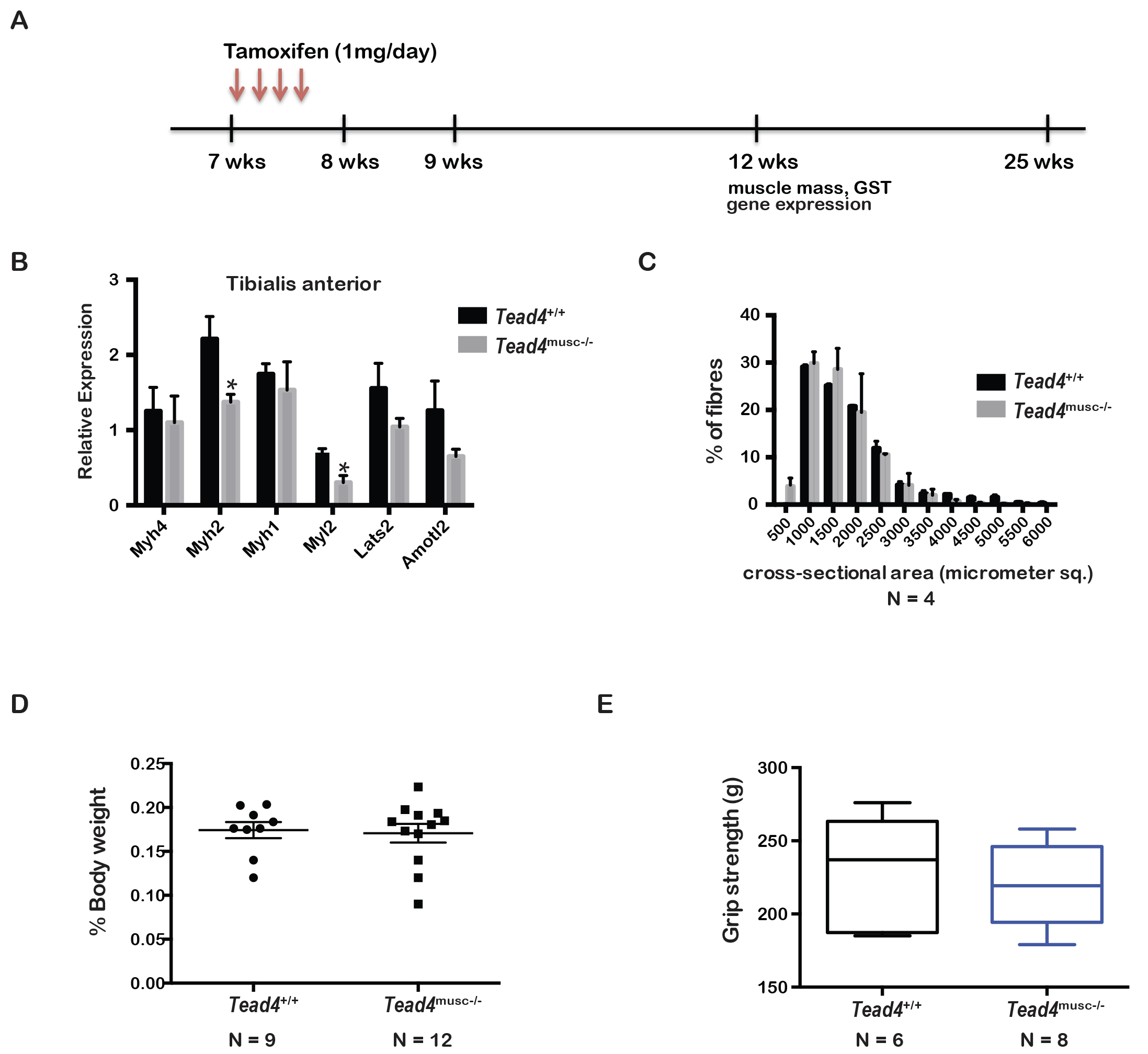

Supplement: S15 Fig — A. Outline of experimental protocol showing days of Tam injection. B. RT-qPCR of the indicated genes. T-test; p-value * <0,05. N = 3. C. Tibialis anterior cross-section area of the indicated animals. Two-way anova, no significant values. N = 4. D. Tibialis anterior mass of the indicated animals. Two-tailed t-test, no-significant values. E. Grip strength of the indicated animals. no-significant values. (TIF) [file pgen.1006600.s015.tif]
